# Targeting UHRF1-SAP30-MXD4 axis for leukemia initiating cell eradication in myeloid leukemia

**DOI:** 10.1038/s41422-022-00735-6

**Published:** 2022-10-27

**Authors:** Cheng-Long Hu, Bing-Yi Chen, Zijuan Li, Tianbiao Yang, Chun-Hui Xu, Ruirui Yang, Peng-Cheng Yu, Jingyao Zhao, Ting Liu, Na Liu, Bin Shan, Qunling Zhang, Junhong Song, Ming-Yue Fei, Li-Juan Zong, Jia-Ying Zhang, Ji-Chuan Wu, Shu-Bei Chen, Yong Wang, Binhe Chang, Dan Hou, Ping Liu, Yilun Jiang, Xiya Li, Xinchi Chen, Chu-Han Deng, Yi-Yi Ren, Roujia Wang, Jiacheng Jin, Kai Xue, Ying Zhang, Meirong Du, Jun Shi, Ling-Yun Wu, Chun-Kang Chang, Shuhong Shen, Zhu Chen, Sai-Juan Chen, Xiaolong Liu, Xiao-Jian Sun, Mingyue Zheng, Lan Wang

**Affiliations:** 1 CAS Key Laboratory of Tissue Microenvironment and Tumor, Shanghai Institute of Nutrition and Health, Shanghai Institutes for Biological Sciences, University of Chinese Academy of Sciences, Chinese Academy of Sciences, Shanghai, China; 2 State Key Laboratory of Medical Genomics, Shanghai Institute of Hematology, National Research Center for Translational Medicine, Ruijin Hospital, Shanghai Jiao Tong University School of Medicine, Shanghai, China; 3 Drug Discovery and Design Center, State Key Laboratory of Drug Research, Shanghai Institute of Materia Medica, Chinese Academy of Sciences, Shanghai, China; 4 School of Pharmaceutical Science and Technology, Hangzhou Institute for Advanced Study, UCAS, Hangzhou, Zhejiang China; 5 Shanghai Institute for Advanced Immunochemical Studies, and School of Life Science and Technology, ShanghaiTech University, Shanghai, China; 6 State Key Laboratory of Cell Biology, Chinese Academy of Sciences Center for Excellence in Molecular Cell Science, Shanghai Institute of Biochemistry and Cell Biology, Chinese Academy of Sciences, University of Chinese Academy of Sciences, Shanghai, China; 7 Key Laboratory of Pediatric Hematology & Oncology of the Ministry of Health of China, Department of Hematology & Oncology, Shanghai Children’s Medical Center, School of Medicine, Shanghai Jiao Tong University, Shanghai, China; 8 School of Pharmacy, Fudan University, Shanghai, China; 9 Department of lymphoma, Fudan University Shanghai Cancer Center, Shanghai, China; 10 Department of Oncology, Shanghai Medical College, Fudan University, Shanghai, China; 11 Shanghai Jiao Tong University School of Life Sciences and Biotechnology, Shanghai, China; 12 Department of Hematology, Shanghai Jiao Tong University Affiliated Sixth People’s Hospital, Shanghai, China; 13 Hospital of Obstetrics and Gynecology, Fudan University Shanghai Medical College, Shanghai, China; 14 Department of Hematology, Shanghai Ninth People’s Hospital, Shanghai Jiao Tong University School of Medicine, Shanghai, China

**Keywords:** Leukaemia, Cell biology

## Abstract

Aberrant self-renewal of leukemia initiation cells (LICs) drives aggressive acute myeloid leukemia (AML). Here, we report that UHRF1, an epigenetic regulator that recruits DNMT1 to methylate DNA, is highly expressed in AML and predicts poor prognosis. UHRF1 is required for myeloid leukemogenesis by maintaining self-renewal of LICs. Mechanistically, UHRF1 directly interacts with Sin3A-associated protein 30 (SAP30) through two critical amino acids, G572 and F573 in its SRA domain, to repress gene expression. Depletion of UHRF1 or SAP30 derepresses an important target gene, *MXD4*, which encodes a MYC antagonist, and leads to suppression of leukemogenesis. Further knockdown of MXD4 can rescue the leukemogenesis by activating the MYC pathway. Lastly, we identified a UHRF1 inhibitor, UF146, and demonstrated its significant therapeutic efficacy in the myeloid leukemia PDX model. Taken together, our study reveals the mechanisms for altered epigenetic programs in AML and provides a promising targeted therapeutic strategy against AML.

## Introduction

Acute myeloid leukemia (AML), a clonal malignancy of abnormal hematopoietic stem/progenitor cells, is characterized by an increase of immature myeloid cells in the bone marrow. AML is the most common acute leukemia in adults, with an incidence of 2.7 per 100,000,^1^ and the 5-year overall survival rate of AML patients remains 25%–40%.^2^ The prognosis is even worse for older patients who are ineligible for intensive chemotherapy and patients with relapsed or refractory AML. Different subtypes of AML including M0-M7^3^ are categorized based on the stage at which normal differentiation is blocked in the leukemic blasts, and the frequently occurred chromosome translocations are also the basis of the improved classification for AML.^4^ Among these translocations, t(8:21) (belongs to M2-subtype AML) that generates the leukemogenic fusion gene AML1-ETO, is the most common translocation in AML.^5,6^ AML with the translocation that leads to MLL gene rearrangements such as MLL-AF9 (belongs to M5-subtype AML) is highly aggressive and resistant to chemotherapy.^7^ Although our understanding of AML has been progressing, chemotherapy remains the main treatment for many AML patients harboring chromosomal translocation. The enhanced self-renewal is an important feature of leukemia initiating cells, which unfortunately could not be effectively targeted by the regular chemotherapy in AML patients. Thus, it is urgent to identify novel therapeutic strategies to target the self-renewal of leukemia initiating cells for AML patients. Some targeted therapeutic drugs for AML, like FLT3 inhibitors Midostaurin and Gilteritinib specifically targeting FLT3 kinase, and isocitrate dehydrogenase (IDH)1/IDH2 inhibitors Ivosidenib and Enasidenib targeting IDH1R132H and 2-hydroxyglutarate respectively, have been tested in clinical trials,^8–11^ whereas studies are still needed to identify more effective targets.

Ubiquitin-like containing PHD Ring Finger 1 (UHRF1), an epigenetic regulator, interacts with DNMT1 directly through the SRA domain, and is required for the maintenance of DNA methylation.^12–16^ Recent studies have shown that UHRF1 plays critical roles in the development of stem/progenitor cells. UHRF1 controls the self-renewal of adult neural stem cells,^17^ and maintains the bivalent histone marks in pluripotent stem cells.^18^ Besides, UHRF1 is essential for sustaining the proliferation of epidermal progenitor cells and suppressing the premature epidermal differentiation.^19^ Moreover, we have found that UHRF1 controls the self-renewal versus differentiation of hematopoietic stem cells (HSCs) by regulating the cell-division modes,^20^ and is required for the invariant natural killer T cell survival and differentiation through the AKT/mTOR signaling pathway.^21^ On the other hand, UHRF1 also regulates cancer development. Overexpression of UHRF1 causes p53-mediated senescence and DNA hypomethylation, and drives hepatocellular carcinoma.^22^ The hemimethylated DNA- and histone-binding functions of UHRF1 support the maintenance of colon cancer oncogenic properties and DNA methylation.^23^ The promoter of *UHRF1* has been shown to be hypomethylated in leukemia patients,^24^ and some studies showed that UHRF1 regulated the viability of acute lymphoid leukemia cell lines in vitro.^25,26^ However, it has not been determined how UHRF1 controls myeloid leukemogenesis in vivo and whether targeting UHRF1 could be an effective therapeutic strategy for the treatment of leukemia.

The epigenetic regulators that play important roles in leukemogenesis are often aberrantly expressed. MLL-AF9 fusion protein has been shown to induce leukemogenesis through abnormal effects on epigenetic regulators (e.g., Dot1L)^27^ and transcriptional modulators (e.g., Meis1, HoxA9, Runx1 and Id1).^28–30^ The AML1-ETO fusion transcription factor also has aberrant transcriptional activation and repression properties.^5,31,32^ We previously identified that AML1-ETO functions in an AML1-ETO-containing complex (AETFC)^33^ and is acetylated by p300 at lysine 43, which is essential for leukemogenesis through activating gene expression.^34^ In our efforts to further explore the mechanism underlying leukemogenesis, we found that UHRF1 is highly expressed in AML patients (including MLL-AF9^+^ and AML1-ETO^+^ AML) compared with the normal subjects, and that high UHRF1 expression is associated with poor prognosis in t(8;21) leukemia patients, independently predicting a shorter event free survival. In this study, we showed that deletion of UHRF1 significantly prolongs the survival time of the mice with AML by targeting the self-renewal of leukemia initiating cells through SAP30-mediated *MXD4* activation. The specific chemical inhibitor of UHRF1, identified by us through library screening, is effective for the AML patient-derived xenograft (PDX) model with low toxicity, which could be a promising therapeutic strategy for AML.

## Results

### UHRF1 is highly expressed in AML and high UHRF1 level predicts poor prognosis

**Fig. 1:**
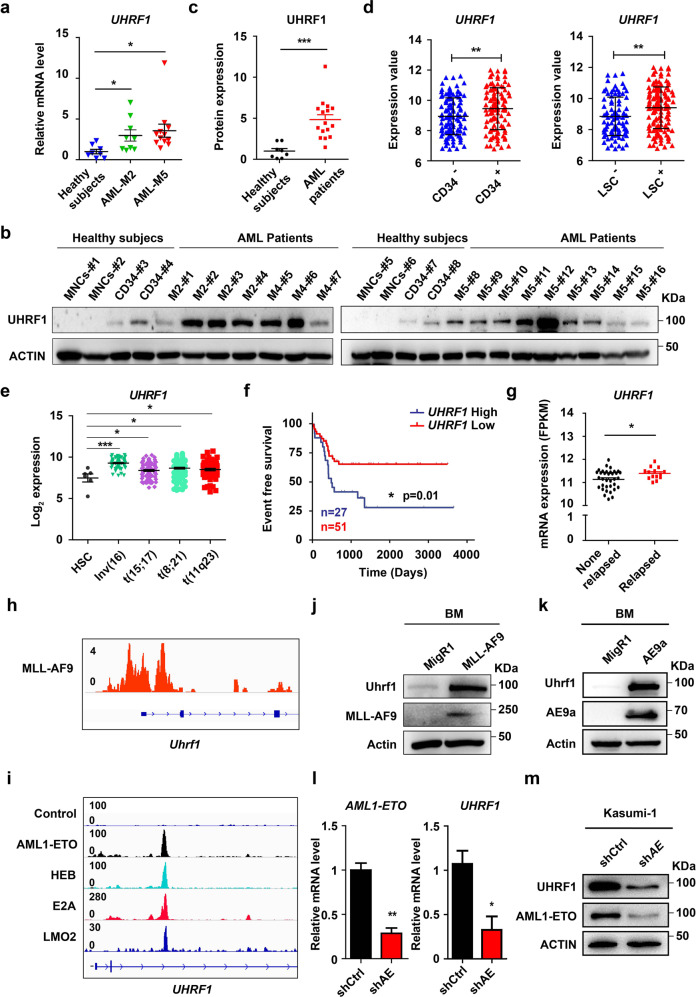
High expression of UHRF1 predicts poor prognosis in AML. **a** The qPCR analysis of *UHRF1* expression in BM mononuclear cells from AML patients (M2, *n* = 9; M5, *n* = 11) and healthy subjects (*n* = 8). **b** The Western blotting analysis of UHRF1 in BM cells of AML patients (*n* = 16), healthy mononuclear cord blood cells (MNCs) (*n* = 4) and CD34^+^ HSPCs (*n* = 4). **c** Quantification of the Western blotting analysis for **b**. **d** The microarray analysis of *UHRF1* in CD34^+^ leukemia cells and LSCs of AML patients from GSE76009. (CD34^+^, *n* = 110; CD34^‒^, *n* = 117; n-LSC^+^, *n* = 138; n-LSC^‒^, *n* = 89). **e** The differential expression of *UHRF1* in mononuclear BM or PB cells of AML patients [*t*(15;17), *n* = 87; Inv(16), *n* = 77; *t*(11q23), *n* = 88; *t*(8;21), *n* = 98)] and HSCs in healthy subjects (HSC, *n* = 6). Data were obtained from the microarray analysis of bloodpool. The samples were normalized and batch corrected using ComBat for full completeness of the dataset. PCA analysis and gene signature values were then calculated. **f** The event free survival of patients with *t*(8;21) leukemia was stratified by *UHRF1* expression into *UHRF1* high (*n* = 27) and low (*n* = 51) groups. The survival days (*UHRF1* high: 510 days; *UHRF1* low: 1067 days) mean the date of the event occurrence in AML patients such as relapse and drug resistance, etc. **g** The differential expression of *UHRF1* in the relapsed (*n* = 14) and non-relapsed (*n* = 35) *t*(8;21) leukemia patients. **h** MLL-AF9 localizes at the promoter region of *Uhrf1* in the CUT&Tag analysis of 32D cells transduced with MLL-AF9. **i** AML1-ETO, HEB, E2A and LMO2 colocalize at the gene body region of *UHRF1* in the ChIP-seq analysis of Kasumi-1 cells. **j**, **k** The mouse BM cells transduced with MLL-AF9 (**j**) or AE9a (**k**) were analyzed. The anti-Uhrf1, anti-MLL1, anti-HA and anti-Actin antibodies were used for the Western blotting analysis. **l**, **m** Knockdown of AML1-ETO in Kasumi-1 cells decreased the mRNA (**l**) and protein (**m**) levels of AML1-ETO and Uhrf1. The anti-Uhrf1, anti-ETO and anti-Actin antibodies were used for the Western blotting analysis. Data are all presented as means ± SD. Statistical analyses were performed using Student’s unpaired *t*-test for **a**, **c**–**e**, **g**, **l**. The expression values for **e** were log_2_ transformed. Statistical significance was evaluated by log-rank test for **f**. The mRNA expression for **g** were statisticed by FPKM from RNA-seq. *P < 0.05; **P < 0.01; ***P < 0.001.

To determine the expression of UHRF1 in AML, we analyzed the bone marrow (BM) samples of AML patients and healthy controls by using RT-qPCR, Western blotting and immunohistochemistry assays. The results showed that the UHRF1 mRNA and protein levels were higher in AML patient samples compared with the healthy controls (Fig. 1a‒c; Supplementary information, Fig. S1a). We then analyzed the international Microarray Innovations in Leukemia (MILE) data,^35^ which showed that *UHRF1* expression is particularly higher in the t(8;21), t(11q23), inv(16) and t(15;17) AML samples relative to healthy HSC controls (Fig. 1e). Moreover, we found that UHRF1 is highly expressed in CD34^+^ AML cells, leukemia stem cells (LSCs)^36^ from AML patients and AML cell lines (Fig. 1d; Supplementary information, Fig. S1b).

As UHRF1 expression is positively correlated with cancer cell proliferation,^37^ it would be interesting to determine how UHRF1 would associate with the prognosis in a specific subtype of AML. To this end, we analyzed our data of t(8;21) AML patients,^38^ and the results showed that the patients with high *UHRF1* expression had a significantly worse event-free survival compared with the patients with low *UHRF1* expression (Fig. 1f). Consistent with this finding, the *UHRF1* expression levels in the relapsed t(8;21) AML patients were significantly higher than those of the non-relapsed patients (Fig. 1g). Thus, these results imply a potential role of UHRF1 as a predictor of prognosis of AML patients.

To further investigate potential mechanism of UHRF1 upregulation in AML, we analyzed our chromatin immunoprecipitation (ChIP)-seq data for AETFC (Kasumi-1 cell) and CUT&Tag data for MLL-AF9 (32D cell). Intriguingly, the results revealed that AETFC components including AML1-ETO (AE), E2A, HEB and LMO2 strongly bind to the gene body of *Uhrf1* (Fig. 1i) and MLL-AF9 also binds to the promoter regions of *Uhrf1* (Fig. 1h). To determine whether Uhrf1 is upregulated in direct response to AE or MLL-AF9, we retrovirally transduced murine BM cells with MIGR1-AE, MIGR1-MLL-AF9 or the MIGR1 vector, and found the elevated Uhrf1 protein in AE or MLL-AF9-transduced cells (Fig. 1j, k). We then used the shRNA against the breakpoint region of *AML1-ETO* to knock down AML1-ETO in Kasumi-1 cells and observed decreased UHRF1 mRNA and protein levels in Kasumi-1 cells with around 70% knockdown (Fig. 1l, m). These data indicate that UHRF1 may be a potential target of the leukemogenic fusion proteins. Together, these results suggest that UHRF1 is highly expressed in AML, and UHRF1 may indicate potential prognosis of AML patients.

### Uhrf1 is required for the maintenance and progression of AML

**Fig. 2:**
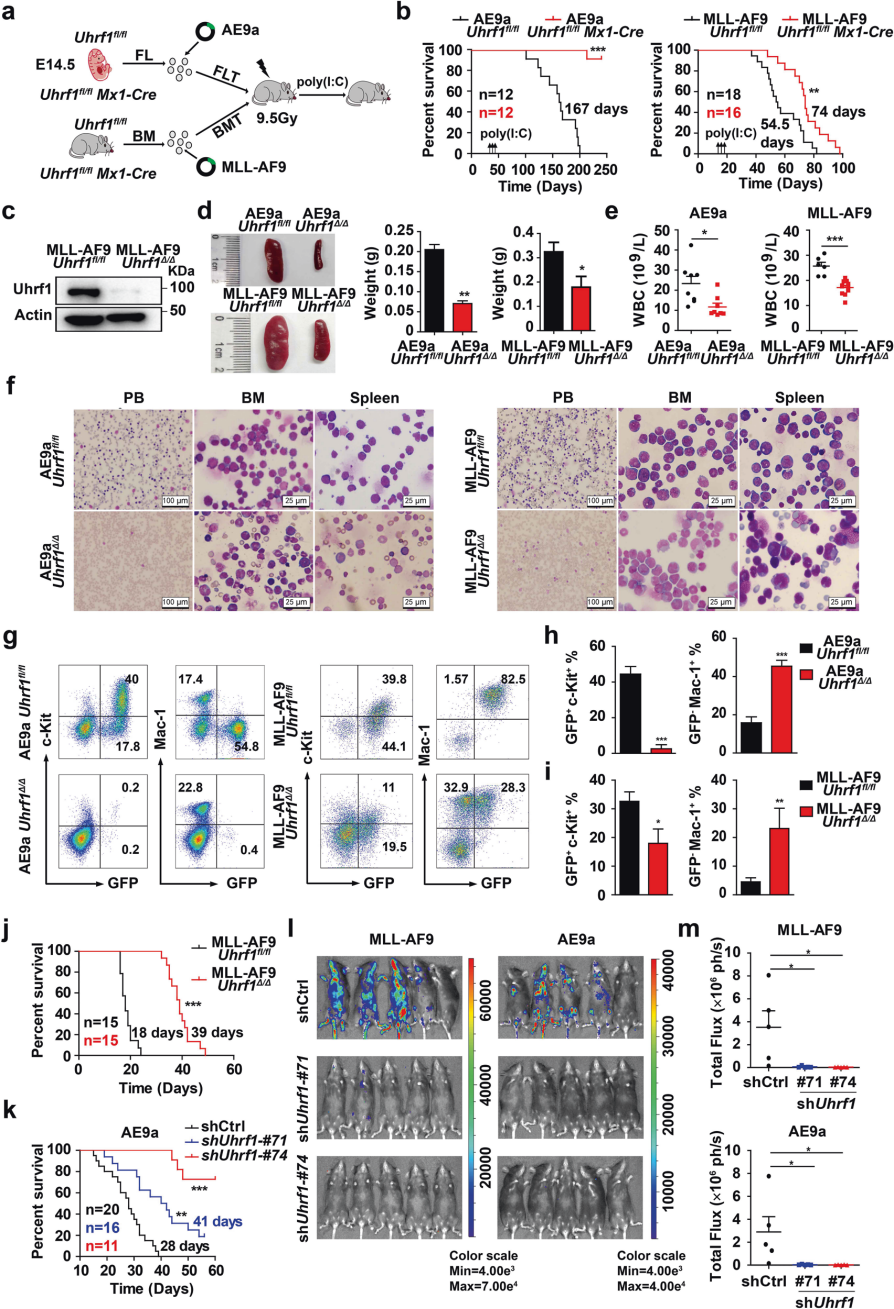
Uhrf1 is required for the maintenance and progression of AML in mouse models of AE9a- or MLL-AF9-driven leukemia. **a** The strategy of AE9a-expressing fetal liver cell transplantation (FLT) or MLL-AF9-expressing BM cell transplantation (BMT). Poly(I:C) was injected to AE9a recipients or MLL-AF9 recipients on week 4 or week 2 respectively. **b** Conditional deletion of *Uhrf1* by poly(I:C) treatment significantly prolongs the survival time of recipient mice transplanted with AE9a*Uhrf1*^*fl/fl*^ Mx1-Cre (*n* = 12) or MLL-AF9*Uhrf1*^*fl/fl*^ Mx1-Cre (*n* ≥ 16) cells. **c** The expression of Uhrf1 is minimal in the sorted GFP^+^ cells isolated from the spleen of the MLL-AF9*Uhrf1*^*fl/fl*^ /MLL-AF9*Uhrf1*^Δ/Δ^ mice. **d** The size and weight of the spleen were decreased in the AE9a*Uhrf1*^Δ/Δ^ and MLL-AF9*Uhrf1*^Δ/Δ^ mice (*n* ≥ 3). **e** The WBC counts of AE9a*Uhrf1*^Δ/Δ^ or MLL-AF9*Uhrf1*^Δ/Δ^ mice were significantly lower than AE9a*Uhrf1*^*fl/fl*^ or MLL-AF9*Uhrf1*^*fl/fl*^ mice (*n* ≥ 6). **f** The PB, BM and spleen show less leukemia blast cells in the AE9a/MLL-AF9*Uhrf1*^Δ/Δ^ mice group compared with the AE9a/MLL-AF9*Uhrf1*^*fl/fl*^ group. **g**‒**i** Representative flow cytometry profiles (**g**) and quantification of the frequencies (**h**, **i**) of GFP^+^c-Kit^+^ leukemia blast cells and normal GFP^−^Mac1^+^ cells in the BM cells of AE9a/MLL-AF9*Uhrf1*^Δ/Δ^ mice compared with AE9a/MLL-AF9*Uhrf1*^*fl/fl*^ mice (*n* ≥ 3). **j** Loss of Uhrf1 significantly prolongs the survival time of recipient mice transplanted with MLL-AF9*Uhrf1*^Δ/Δ^ cells compared with MLL-AF9*Uhrf1*^*fl/fl*^ group (*n* = 15). **k** Knockdown of Uhrf1 significantly prolongs the survival time of recipient mice transplanted with AE9a cells, compared with the control shRNA (*n* ≥ 11). **l**, **m** The in vivo bioluminescence imaging (**l**) and quantification analysis (**m**) shows that knocking down *Uhrf1* impairs the leukemia progression in recipient mice transplanted with AE9a or MLL-AF9 leukemia cells that are luciferase-positive (*n* = 5). Data are all presented as means ± SD. Statistical significance was evaluated by log-rank test for **b**, **j** and **k**. Statistical analyses were performed using Student’s unpaired *t*-test for **e**, **h**, **i**, **m**. *P < 0.05, **P < 0.01, ***P < 0.001.

To investigate the role of Uhrf1 in leukemia maintenance, we utilized *Uhrf1* conditional knockout mice for AML studies. We isolated the E14.5 fetal liver (FL) cells and adult BM cells from *Uhrf1*^*fl*/*fl*^ and *Uhrf1*^*fl/fl*^ *Mx1*-Cre mice, which were transduced with the retrovirus expressing AML1-ETO9a (AE9a) or MLL-AF9, respectively. The lethally irradiated recipient mice were injected with these transduced cells via tail vein (Fig. 2a), and poly(I:C) was injected to the recipients to induce *Uhrf1* deletion. The Western blotting analysis showed that Uhrf1 was significantly depleted in the leukemia cells of *Uhrf1*^*fl/fl*^ *Mx1*-Cre recipients upon the poly(I:C) administration (Fig. 2c). The recipients receiving AE9a-transduced *Uhrf1*^*fl/fl*^ *Mx1*-Cre FL cells failed to develop AML post-poly(I:C) induction, while the control recipients develop AML with a latency of 167 days (Fig. 2b). In the mouse model of MLL-AF9-driven leukemia, the MLL-AF9*Uhrf1*^Δ/Δ^ mice showed a significantly longer median survival (74 days) as compared with the MLL-AF9*Uhrf1*^*fl/fl*^ mice (54.5 days; Fig. 2b). The AE9a/MLL-AF9*Uhrf1*^Δ/Δ^ mice had significantly smaller spleen (Fig. 2d), and lower white blood cell (WBC) counts as compared with AE9a/MLL-AF9*Uhrf1*^*fl/fl*^ mice (Fig. 2e). Meanwhile, the Wright’s staining analysis revealed lots of leukemia blasts emerged in the peripheral blood (PB), bone marrow (BM) and spleen cells of AE9a/MLL-AF9*Uhrf1*^*fl/fl*^ mice, whereas normal components of hematopoietic cells in AE9a*Uhrf1*^Δ/Δ^ mice and less leukemia blasts in MLL-AF9*Uhrf1*^Δ/Δ^ mice were shown (Fig. 2f). Similarly, the histology analysis indicated that the AE9a/MLL-AF9*Uhrf1*^Δ/Δ^ mice had less infiltration of leukemia in the BM, spleen and liver compared with AE9a/MLL-AF9*Uhrf1*^*fl/fl*^ mice (Supplementary information, Fig. S2a, b). The flow cytometry analysis showed that BM cells of AE9a/MLL-AF9*Uhrf1*^Δ/Δ^ mice contained far fewer GFP^+^c-Kit^+^ leukemia blast cells and more GFP^‒^Mac-1^+^ (i.e., normal) cells compared with AE9a/MLL-AF9*Uhrf1*^*fl/fl*^ mice (Fig. 2g‒i).

To further identify the role of Uhrf1 in AML progression, we transplanted 1 × 10^5^ MLL-AF9-expressing *Uhrf1*^*fl/fl*^ or *Uhrf1*^Δ/Δ^ leukemia cells into sublethally irradiated recipient mice. *Uhrf1* deficiency strikingly prolonged the median survival of the secondarily transplanted recipients (39 days vs 18 days) (Fig. 2j). Two weeks after transplantation, the WBC counts of the MLL-AF9*Uhrf1*^Δ/Δ^ recipients were significantly lower than those of the MLL-AF9*Uhrf1*^*fl/fl*^ recipients, and the red blood cell (RBC) counts, platetlet (PLT) counts and hemoglobin (HGB) concentrations were higher, reflecting the impaired leukemogenesis (Supplementary information, Fig. S2c). The splenomegaly and hepatomegaly were prominently observed in the MLL-AF9*Uhrf1*^*fl/fl*^ recipients, but were less in the MLL-AF9*Uhrf1*^Δ/Δ^ recipients (Supplementary information, Fig. S2d, e). The flow cytometry analysis showed that nearly 90% of BM cells of *Uhrf1*^*fl/fl*^ recipients were GFP^+^Gr-1^+^ or GFP^+^Mac-1^+^, but only 10% of BM cells in *Uhrf1*^Δ/Δ^ recipients were GFP^+^Gr-1^+^ or GFP^+^Mac-1^+^ (Supplementary information, Fig. S2f). Meanwhile, the peripheral blood smear analysis, the cytospin analysis of the BM and spleen cells (Supplementary information, Fig. S2g), and the histology analysis of the BM, SP, and liver (Supplementary information, Fig. S2h) revealed less leukemic blasts in the MLL-AF9*Uhrf1*^Δ/Δ^ recipients than the MLL-AF9*Uhrf1*^*fl/fl*^ recipients. We also sought to knock down *Uhrf1* in AE9a or MLL-AF9 cells and evaluate their growth potential in the sublethally irradiated recipients. The mice receiving the sh*Uhrf1*-expressing AE9a or MLL-AF9 cells had a longer life span than the mice receiving scrambled shRNA-transduced cells (Fig. 2k; Supplementary information, Fig. S3a, b). These mice also had smaller spleen size, suggesting the leukemogenicity of AE9a or MLL-AF9 leukemia cells was reduced by *Uhrf1* knockdown in vivo (Supplementary information, Fig. S3c‒f). Since these cells are luciferase-positive, we detected more intense bioluminescent signals in the control mice compared with the recipients receiving the sh*Uhrf1*-expressing AE9a or MLL-AF9 cells (Fig. 2l, m). Together, these results suggest that Uhrf1 is required for the maintenance and progression of AML.

### Uhrf1 deficiency impairs the self-renewal of leukemia initiating cells (LICs) and decreases the frequency of LICs

**Fig. 3:**
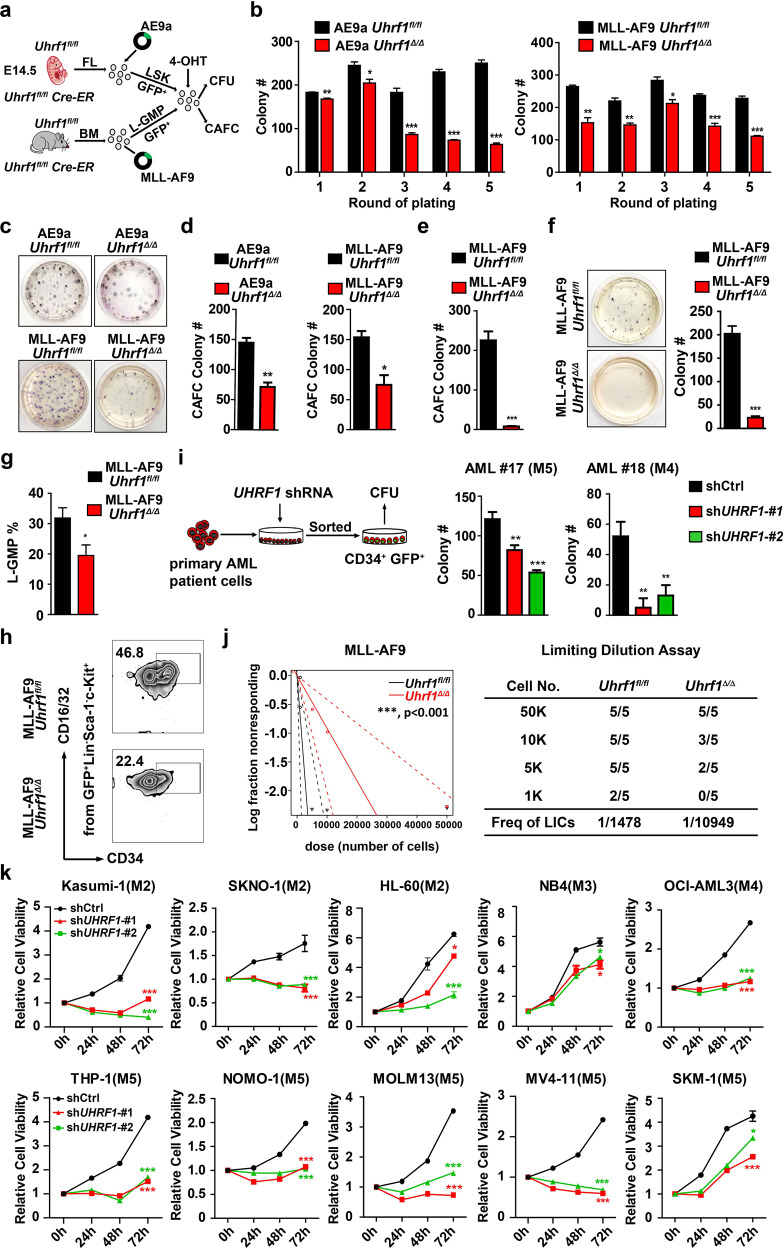
Loss of Uhrf1 impairs the self-renewal of LICs and decreases the frequency of LICs. **a** The strategy of analysis of AE9a- and MLL-AF9-driven LICs. LSK cells represent LIC cells in AE9a-driven AML (Lin–Sca-1^+^c-Kit^+^), and L-GMP cells represent LICs in MLL-AF9-driven AML (Lin^−^Sca-1^−^c-Kit^+^CD34^+^CD16/32^+^ GMP-like leukemic cells). **b**, **c** The average number of colonies (**b**) generated from 3000 AE9a-expressing FL LSK cells or 800 MLL-AF9-expressing BM L-GMP cells with or without *Uhrf1* deletion upon 4-OHT treatment in each replating (*n* = 3), and the morphology of the colonies (**c**). **d** Conditional deletion of *Uhrf1* by 4-OHT treatment decreases the self-renewal capacity of AE9a- or MLL-AF9-driven LICs in Long-Term Culture-Initiating Cell (LTC-IC) assays. Shown is the CAFC numbers of colonies generated from 4000 AE9a-expressing FL LSK cells or 500 MLL-AF9-expressing BM L-GMP cells (*n* = 3). **e** The CAFC numbers of colonies generated from 3000 MLL-AF9*Uhrf1*^*fl/fl*^ or MLL-AF9*Uhrf1*^Δ/Δ^ leukemia blast cells from recipients in the primary transplantation (*n* = 3). **f** The morphology and numbers of colonies generated from 2000 MLL-AF9*Uhrf1*^*fl/fl*^ or MLL-AF9*Uhrf1*^Δ/Δ^ leukemia blast cells from recipients in the primary transplantation (*n* = 3). **g**, **h** Representative flow cytometry profiles (**h**) and quantification of the frequencies (**g**) of L-GMP cells in the BM from MLL-AF9*Uhrf1*^*fl/fl*^ or MLL-AF9*Uhrf1*^Δ/Δ^ recipients (*n* ≥ 5). **i** The average numbers of colonies generated from 10,000 primary AML CD34^+^ patient cells with *UHRF1* knockdown by shRNA (*n* = 3). **j** The log-fraction plot shows the result of the limiting dilution assay by using different dilutions of leukemia cells from MLL-AF9*Uhrf1*^*fl/fl*^ or MLL-AF9*Uhrf1*^Δ/Δ^ mice. The “solid line” means confidence of intervals for 1/LICs of estimate, and “dotted line” means confidence of intervals for 1/LICs of lower and upper. **k** The MTT assays show that knocking down *UHRF1* significantly inhibited the proliferation of AML cells (*n* = 3). Statistical analyses were performed using Student’s unpaired *t*-test for **b**, **d**–**g**, **i** and **k**. ELDA software was used for analysis in **j**. Data are all presented as means ± SD; *P < 0.05, **P < 0.01, ***P < 0.001.

To explore the role of Uhrf1 in self-renewal of LICs, we crossed the Cre-ER transgenic mice with *Uhrf1*^*fl/fl*^ mice to generate *Uhrf1*^*fl/fl*^ Cre-ER mice. E14.5 FL or adult BM cells of *Uhrf1*^*fl/fl*^ Cre-ER mice were transduced with AE9a or MLL-AF9 (MA-9) and LICs were sorted to the culture medium (Fig. 3a). We used 1 µM 4-hydroxy-tamoxifen (4-OHT) to induce *Uhrf1* deletion in vitro and the Western blotting analysis showed that Uhrf1 has been almost totally deleted (Supplementary information, Fig. S3g). We performed the serial replating colony formation assay to evaluate the self-renewal of LICs. The results revealed that loss of *Uhrf1* significantly decreased the number of colonies (Fig. 3b, c). To further study the long-term self-renewal ability of LICs, we conducted the cobble stone area formation colony assay (CAFC) and found that loss of *Uhrf1* significantly decreased the number of cobble stone area colonies (Fig. 3d). *Uhrf1* deficiency significantly decreased the number of CFU and CAFC colonies derived from the mouse MLL-AF9-expressing c-Kit^+^ leukemia blast cells (Fig. 3e, f). We performed flow cytometry analysis of LICs in BM cells of recipients with MLL-AF9-driven AML 4 weeks after *Uhrf1* deletion by poly(I:C) administration, and found that *Uhrf1* deletion significantly decreased the percentage of LICs from the recipient mice in the primary transplantation (Fig. 3g, h). Moreover, knocking down *UHRF1* significantly decreased the number of colonies derived from human CD34^+^ BM cells of AML patients in CFU assay (Fig. 3i). These results suggest that Uhrf1 deficiency impairs the self-renewal of LICs.

To directly enumerate the frequency of LICs, we performed the limiting dilution assay of MLL-AF9- or AE9a-driven leukemia. The results showed that Uhrf1 deficiency decreased the frequency of LICs in MLL-AF9- or AE9a-driven AML mice (1:10,949 vs 1:1478 cells and 1:3374 vs 1:747 cells, respectively) (Fig. 3j; Supplementary information, Fig. S3h). In summary, our data indicate that Uhrf1 is essential for the self-renewal of LICs.

### Knocking down *UHRF1* affects the survival and cell cycle of AML cells

To define the function of UHRF1 in AML cells, we knocked down *UHRF1* in AML cell lines by using shRNA against *UHRF1* and found that inhibition of *UHRF1* significantly inhibited the growth of these cells (Fig. 3k; Supplementary information, Fig. S4a, b). The flow cytometry and morphology analysis showed that knockdown of *UHRF1* significantly induced the apoptosis of Kasumi-1 and THP-1 cells (Supplementary information, Fig. S4c‒e). After knocking down *UHRF1*, we found the increased BAX and decreased PARP in these cells (Supplementary information, Fig. S4f), and the expansion of AML cells were reduced in CFU assay (Supplementary information, Fig. S4g, h). The flow cytometry results indicated that AML cells were significantly arrested at G2/M phase and the Western blotting results showed that the level of cell cycle-related protein p27 was increased after *UHRF1* knockdown (Supplementary information, Fig. S4f, I, j).

We further studied the role of Uhrf1 in the murine AML cells. The CFU assay analysis showed that the expansion of AE9a or MLL-AF9 cells also decreased after knocking down *Uhrf1* (Supplementary information, Fig. S3i, j). Moreover, the flow cytometry analysis showed that AE9a or MLL-AF9 cells were significantly arrested at G2/M phase after knocking down *Uhrf1* (Supplementary information, Fig. S3k, l). In summary, these results strongly suggest that UHRF1 is essential for the proliferation and survival of AML cells.

### The genome-wide DNA binding patterns of UHRF1 in leukemia cells and transcriptome change induced by UHRF1 knockdown

**Fig. 4:**
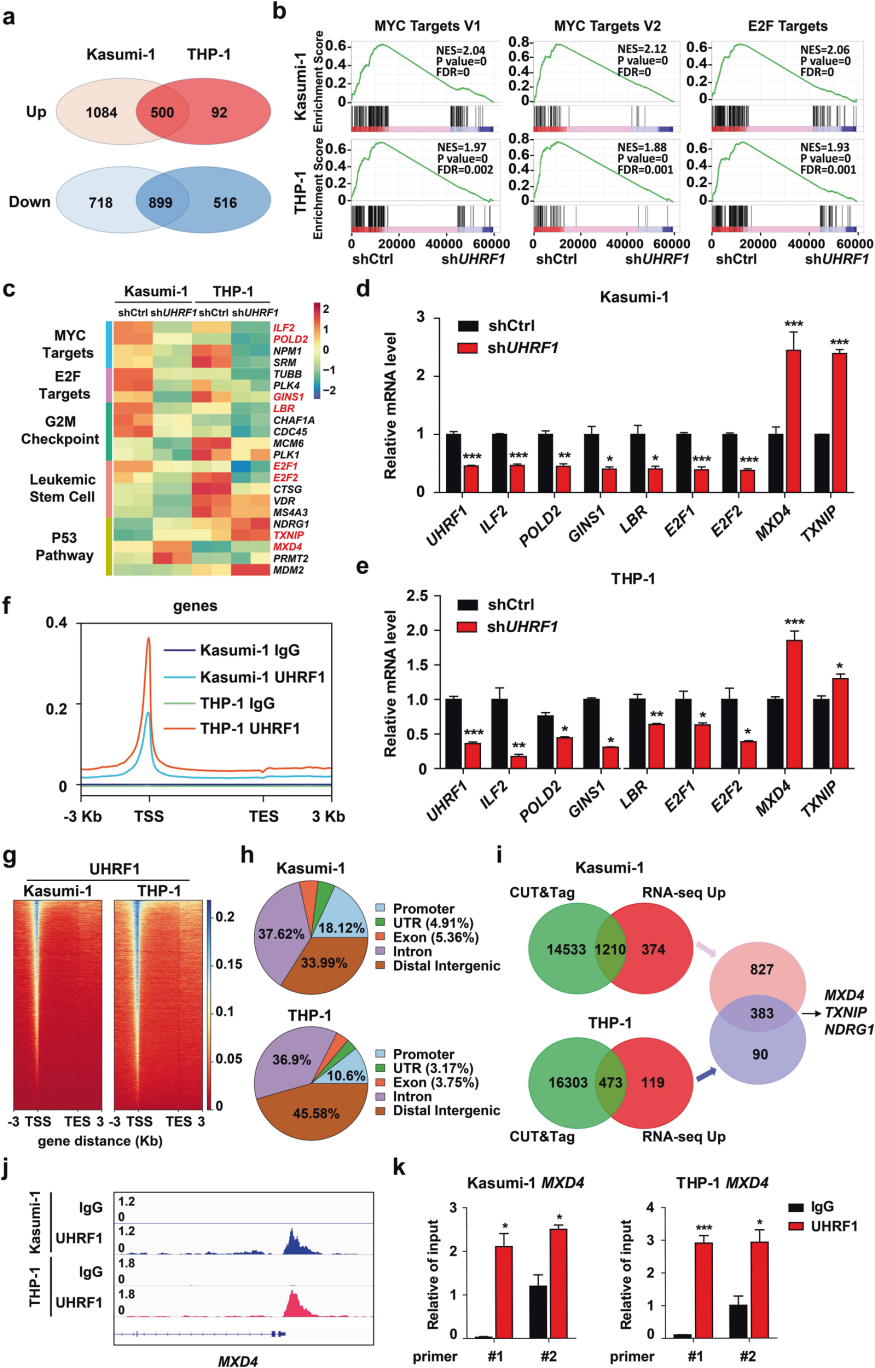
The genome-wide DNA binding patterns of UHRF1 and low expression of UHRF1 changes the transcriptome in Kasumi-1 and THP-1 cells. **a** RNA-seq was performed on Kasumi-1 and THP-1 cells with *UHRF1* knockdown, and the overlap of DEGs in RNA-seq was shown. **b** The GSEA curves for the pathways involving MYC and E2F targets selected from the top 10 affected pathways in AML cells with *UHRF1* knockdown. **c** The heat map analysis of the enriched pathways in RNA-seq data of AML cells with *UHRF1* knockdown. **d**, **e** The expression of genes in the enriched pathways was examined by q-PCR analysis in Kasumi-1 (**d**) and THP-1 (**e**) cells with *UHRF1* knockdown (*n* = 3). **f** The CUT&Tag analysis was performed with the anti-UHRF1 antibody and control IgG in AML cells, and a profile of UHRF1 binding, centering on the TSS, was shown. **g** The heat-maps of CUT&Tag peak signals of UHRF1 target genes in AML cells. **h** The distribution of UHRF1 binding sites in AML cells by the CUT&Tag analysis. **i** The overlap of UHRF1 target genes from the CUT&Tag analysis and DEGs from RNA-seq analysis shows 383 common genes regulated by UHRF1 in both Kasumi-1 and THP-1 cells. **j** The binding peak of UHRF1 was identified on *MXD4* promoter based upon the CUT&Tag analysis in AML cells. **k** The ChIP-qPCR analysis of UHRF1 binding to the *MXD4* loci in AML cells. Data are all presented as means ± SD. Statistical analyses were performed using Student’s unpaired *t*-test for **d**, **e**, and **k**. *P < 0.05, **P < 0.01, ***P < 0.001.

To investigate how UHRF1 regulates leukemogenesis, we performed the RNA-seq analysis of AML cells with or without UHRF1 knockdown. The result showed that 500 genes and 899 genes were significantly up-regulated or down-regulated in both Kasumi-1 and THP-1 cells after UHRF1 knockdown by analysis of the overlapped differential expression genes (DEGs) (Fig. 4a; Supplementary information, Fig. S5a). Gene ontology (GO) analysis of DEGs showed that GO terms or pathways such as the myeloid cell homeostasis, hemi-methylated DNA-binding, cell growth, apoptotic signaling, cell cycle-related pathways are highly enriched in AML cells upon UHRF1 knockdown (Supplementary information, Fig. S5b). The gene set enrichment analysis (GSEA) revealed that genes sets related to the MYC targets, E2F targets, G2/M checkpoint, leukemia stem cell and p53 pathway are highly enriched (Fig. 4b, c; Supplementary information, Fig. S7o). Some genes that were dysregulated in AML cells with UHRF1 knockdown (e.g., *MXD4*, *E2F1*, *E2F2*, *ILF2*, *LBR, POLD2, GINS1* and *TXNIP*) play vital roles in regulating these pathways (Fig. 4c). The mRNA levels of some of these genes form the enriched pathways were verified by the q-PCR analysis (Fig. 4d, e).

To define the direct targets of UHRF1, we performed the CUT&Tag analysis by using the anti-UHRF1 antibody and IgG control in AML cells. The profile and heat-map of signal peaks showed that the binding sites of UHRF1 on genes are mainly at transcription start sites (TSS) (Fig. 4f, g), distal intergenic regions, intron, promoters (Fig. 4h) and up-stream/down-stream of TSS (Supplementary information, Fig. S5c). To further identify the role of UHRF1-regulated target genes in leukemia cells, we analyzed the DEGs from RNA-seq combined with the CUT&Tag data and found that there were 383 overlapped target genes in AML cells including *MXD4* that encodes the transcriptional repressor MXD4, an antagonist of MYC^39,40^ (Fig. 4i). Altogether, these results showed that UHRF1 deficiency led to decreased transcription of the MYC signaling pathway-related genes and increased expression of *MXD4* in AML cells.

### UHRF1-mediated MXD4 repression is essential for leukemogenesis

**Fig. 5:**
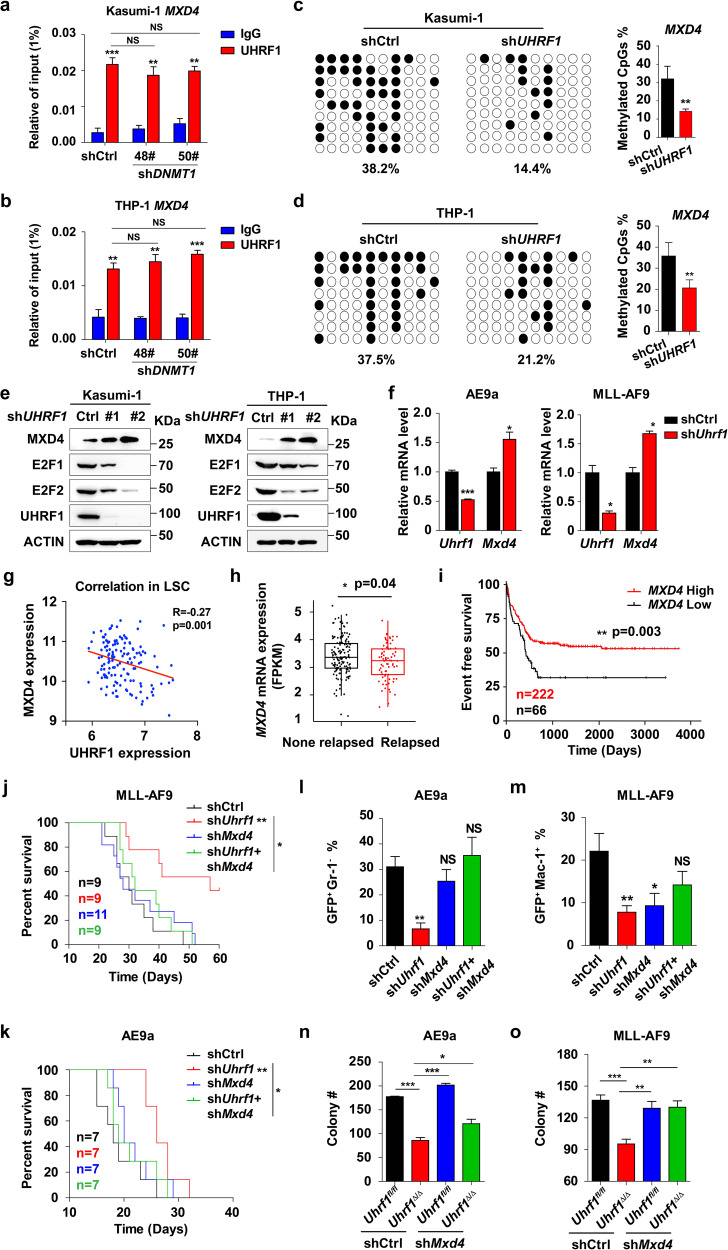
Repression of MXD4 expression by UHRF1 is essential for leukemogenesis. **a**, **b** ChIP-qPCR analysis of the TSS enrichment of UHRF1 on *MXD4* gene in Kasumi-1 (**a**) and THP-1 (**b**) cells with DNMT1 knockdown by using the anti-UHRF1 antibody. **c**, **d** The DNA methylation analysis of *MXD4* by the bisulfite sequencing in Kasumi-1 (**c**) and THP-1 (**d**) cells with UHRF1 knockdown (*n* = 3). **e** The protein levels of MXD4 and E2F1 were examined by Western blotting analysis in AML cells with UHRF1 knockdown. **f** The expression of *Mxd4* was examined by q-PCR analysis in murine AML cells with *Uhrf1* knockdown (*n* = 3). **g** Correlation analysis of UHRF1/MXD4 expression in LSCs from AML patients was performed using the GSE76009 dataset. **h** RNA-seq analysis shows that the expression of *MXD4* is lower in the relapsed AML patients (*n* = 77) compared with the non-relapsed AML patients (*n* = 146). **i** The event free survival of AML patients was stratified by *MXD4* expression into *MXD4-*high (679 days, *n* = 222) and *MXD4-*low (405 days, *n* = 66) groups. **j**, **k** The survival of recipient mice receiving AE9a (*n* = 7) (**j**) or MLL-AF9 (*n* = 6) (**k**) cells with the simultaneous knockdown of *Uhrf1* and *Mxd4*. **l**, **m** The flow analysis of GFP, Mac-1 and Gr-1 in BM cells of the recipient mice in AE9a (**l**) or MLL-AF9 (**m**) mouse model (*n* ≥ 3). **n**, **o** The number of colonies generated from AE9a (**n**) or MLL-AF9 (**o**) driven LICs with *Uhrf1* deletion/*Mxd4* knockdown (*n* = 3). Data are all presented as means ± SD. Statistical analyses were performed using Student’s unpaired *t*-test for **a**–**d**, **f**, **h**, **l**–**o**, and log-rank test for **i**–**k**. Pearson’s correlation analysis was used for **g**. *P < 0.05, **P < 0.01, ***P < 0.001.

To assess functionally important target genes of UHRF1, we analyzed the CUT&Tag data and validated some binding events by ChIP-qPCR in AML cells. The CUT&Tag and ChIP-qPCR data showed that UHRF1 directly bound to *MXD4* promoter (Fig. 4j, k). To determine whether the TSS enrichment of UHRF1 is DNA methylation independent, we performed ChIP assay in AML cells with DNMT1 knockdown by using an antibody against UHRF1 (Supplementary information, Fig. S5d). The results showed that the enrichment of UHRF1 on *MXD4* promoter was not significantly changed in AML cells with DNMT1 knockdown compared with the control cells (Fig. 5a, b). We surveyed the overlapped dysregulated genes of RNA-seq and CUT&Tag data and found that *MXD4* was significantly upregulated by UHRF1 knockdown in murine AML and LICs (Fig. 5e, f; Supplementary information, Fig. S5e, f). Given the vital role of UHRF1 in DNA methylation, we performed bisulfite sequencing of *MXD4* to test the DNA methylation level of *MXD4* and found that knocking down UHRF1 decreased the level of *MXD4* DNA methylation at the CpG sites around the TSS in AML cells (Fig. 5c, d). We found that the expression of UHRF1 is negatively correlated with the expression of MXD4 in LSCs from AML patients by using microarray analysis (GSE76009) (Fig. 5g). These data suggest that UHRF1 inhibition-induced activation of *MXD4* transcription is possibly due to the decreased DNA methylation. Thus, we identified *MXD4* as a target gene of UHRF1 based on RNA-seq, CUT&Tag and bisulfite sequencing analyses.

To examine whether *MXD4* could predict the clinical outcomes of AML patients, we analyzed 288 patients with AML by performing RNA-seq of the leukemic cells isolated from their BM, and the result showed that *MXD4* expression is significantly lower in the relapsed patients (Fig. 5h). The Kaplan-Meier curves show that the patients with low *MXD4* expression have a significantly higher chance of relapse (Fig. 5i). Therefore, these data indicate that low expression of *MXD4* is associated with a high relapse rate of AML patients.

It was known that MYC binding with MAX stimulates the cell cycle progression and cell proliferation through regulation of E2F, and that MXD4 can inhibit MYC function by competing with MAX binding.^39,41^ We indeed found that E2F is decreased in AML cells with *UHRF1* knockdown by the Western blotting analysis (Fig. 5e). To understand the role of MXD4 in leukemogenesis, we sought to knock down both *Mxd4* and *Uhrf1* in AE9a or MLL-AF9 cells (Supplementary information, Fig. S5g) and found that knockdown of Mxd4 significantly rescued Uhrf1 deficiency-induced apoptosis in AE9a and MLL-AF9 cells (Supplementary information, Fig. S7a, b). We also evaluated the growth potential of these cells in sublethally irradiated recipient mice. The recipients receiving the sh*Mxd4* and sh*Uhrf1*-expressing AE9a or MLL-AF9 cells had a shorter life span than the mice who received sh*Uhrf1*-transduced cells (Fig. 5j, k). These mice also had more AE9a- or MLL-AF9-expressing cells (GFP^+^) in their PB two weeks after the transplantation, demonstrating that the delayed leukemogenesis of sh*Uhrf1*-expressing AE9a or MLL-AF9 cells was restored by Mxd4 knockdown in vivo (Fig. 5l, m; Supplementary information, Fig. S5h). Knocking down Mxd4 also rescued *Uhrf1* deficiency-disrupted self-renewal of AE9a or MLL-AF9-expressing LICs in the CFU assay (Fig. 5n, o).

### The interaction of UHRF1 and SAP30 is required for *MXD4* repression and leukemogenesis

**Fig. 6:**
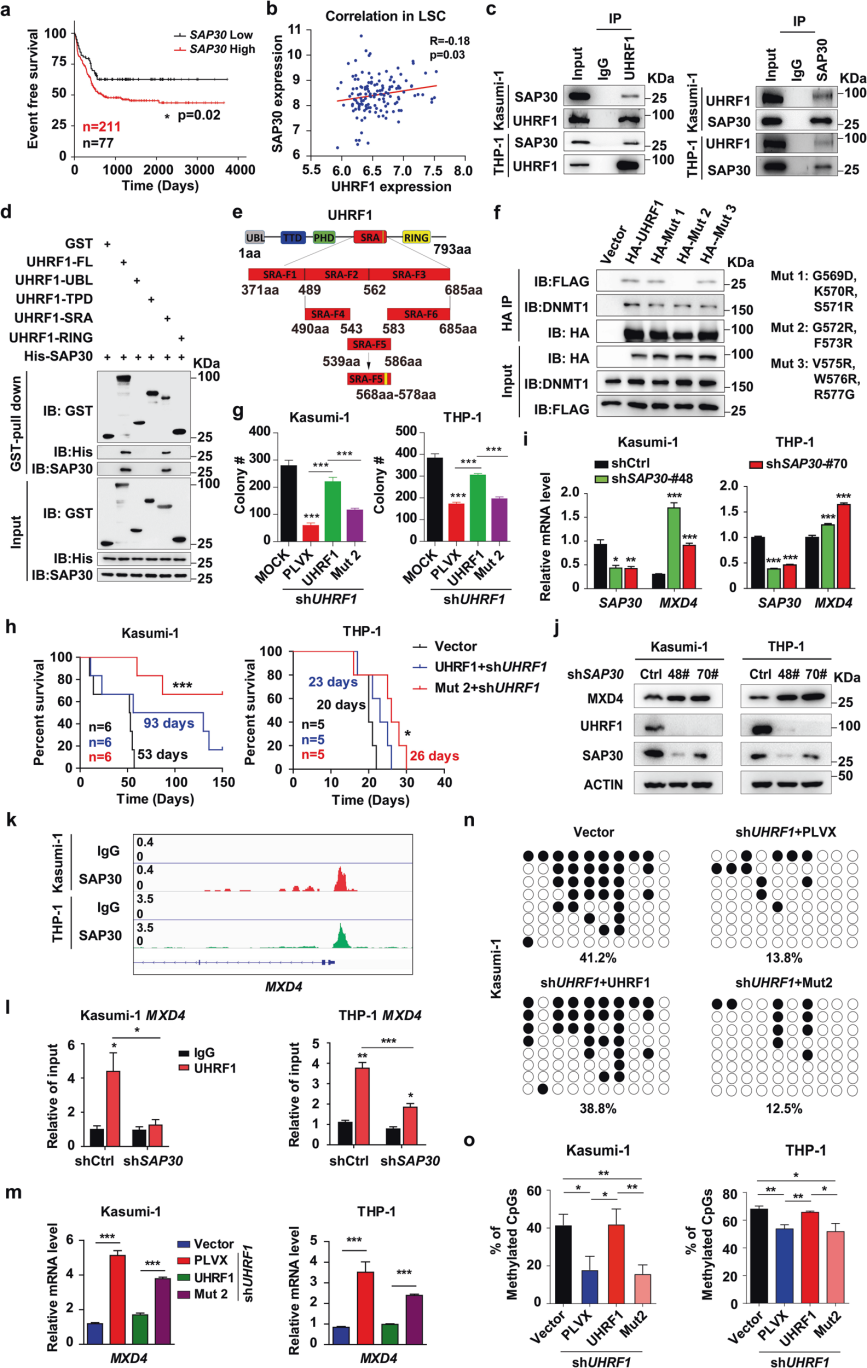
The interaction of UHRF1 with SAP30 is critical for MXD4 repression and leukemogenesis. **a** The event free survival of AML patients was stratified by *SAP30* expression into *SAP30* high (survival days: 503 days, *n* = 211) and low (survival days: 646 days, *n* = 77) groups. **b** Correlation analysis of UHRF1/SAP30 expression in LSCs from AML patients was performed using the GSE76009 dataset. **c** The immunoprecipitation was performed using the anti-UHRF1 or anti-SAP30 antibody, and the anti-SAP30 and anti-UHRF1 antibodies were used for the Western blotting analysis in AML cells (*n* ≥ 3). **d** The GST pull-down assay shows that UHRF1 interacts with SAP30 in vitro and the SRA domain of UHRF1 is required for the interaction with SAP30. **e** The schematic representation of the truncations of SRA domain. **f** The immunoprecipitation assay was performed to examine the interaction of HA-tagged mutant UHRF1 with Flag-SAP30 in 293T cells (*n* = 3). **g** The number of colonies generated from UHRF1-deficient AML cells transduced with WT or mutant UHRF1 (*n* = 3). **h** The survival of B-NDG recipient mice receiving Kasumi-1 (*n* = 6) or THP-1 (*n* = 5) cells with the restoration of UHRF1 and UHRF1-Mut2 after UHRF1 knockdown. **i**, **j** *MXD4* expression was examined by q-PCR (**i**) and Western blotting (**j**) analysis in AML cells with SAP30 knockdown (*n* = 3). **k** The CUT&Tag analysis shows that SAP30 binds to the promoter of *MXD4* in AML cells. **l** The ChIP-qPCR analysis of UHRF1 on the promoter of *MXD4* in AML cells with the knockdown of SAP30 (*n* = 3). **m** *MXD4* expression was examined by q-PCR analysis in UHRF1-deficient AML cells transduced with WT or mutant UHRF1 (*n* = 3). **n**, **o** The representative DNA methylation profiles (**n**) and quantification (**o**) analysis of *MXD4* by the bisulfite sequencing in G572R and F573R mutant AML cells with UHRF1 knockdown. Data are all presented as means ± SD. Statistical analyses were performed using log-rank test for **a**, **h**, and Pearson’s correlation analysis for **b**. Student’s unpaired *t*-test was used for **g**, **i**, **l**, **m**, **o**. *P < 0.05, **P < 0.01, ***P < 0.001.

To further understand the role of UHRF1 in leukemogenesis, we examined the proteins interacting with UHRF1 in AML cells by the mass spectrometry analysis and identified that UHRF1 bound to Sin3A-associated protein (SAP30), which mediates protein‒DNA interactions and is involved in transcriptional regulation (Supplementary information, Fig. S6a, b).^42^ Analysis of clinical samples showed that *SAP30* was highly expressed in AML patients^35,43^ and AML cell lines (Supplementary information, Fig. S6c, d), and the high expression of *SAP30* predicts the poor event-free survival in AML (Fig. 6a). The expression of SAP30 was positively correlated with the expression of UHRF1 in LSCs from AML patients (Fig. 6b). We verified that UHRF1 interacts with SAP30 by using the Co-immunoprecipitation (Co-IP) assays in AML cells (Fig. 6c). Next, we performed the glutathione S-transferase (GST) pull-down assay and found a direct interaction between UHRF1 and SAP30 through the SRA domain (Fig. 6d; Supplementary information, Fig. S6e). To further identify the interaction site of UHRF1 and SAP30, we truncated the SRA domain into three segments, and the GST pull-down analysis showed that SRA-F2 and SRA-F3 directly interacted with SAP30 (Supplementary information, Fig. S6f, g). Then, we constructed the UHRF1 plasmid with different truncated fragments in the SRA-F2 and SRA-F3 regions (Supplementary information, Fig. S6h, i) and identified that the truncation of an 11-amino acid fragment (aa568‒aa578) in the SRA domain blocked the interaction between UHRF1 and SAP30 by the Co-IP assay (Fig. 6e; Supplementary information, Fig. S6j). Finally, we performed a screening of mutation in the aa568‒aa578 fragment and identified the point mutations G572R and F573R that specifically disrupt the UHRF1‒SAP30 interaction but not the UHRF1‒DNMT1 binding, as indicated by the Co-IP assay (Fig. 6f). The G572 and F573 residues are highly conserved in other species (Supplementary information, Fig. S6k), suggesting that this interaction may be evolutionary conserved.

To explore the biological relevance of UHRF1‒SAP30 interaction in leukemogenesis, we expressed UHRF1 and the UHRF1-Mut2 mutant in AML cells with UHRF1 suppression (shUHRF1 AML cells) for the CFU assay. Although UHRF1 and the Mut2 mutant exhibited comparable expression levels in the transduced AML cells, Mut2 significantly impaired the ability of UHRF1 to rescue the self-renewal activity of shUHRF1 AML cells (Fig. 6g; Supplementary information, Fig. S6l). To determine whether the UHRF1‒SAP30 interaction is required for leukemogenesis in vivo, we used the cell-derived xenograft (CDX) model based on the transplantation of shUHRF1 AML cells transduced with the wild type UHRF1 or mutated UHRF1. In this assay, the mice carrying UHRF1-Mut2 showed a significant delay in leukemogenesis compared with the control mice (Fig. 6h), indicating that the UHRF1‒SAP30 interaction is required for acute myeloid leukemogenesis.

To determine whether SAP30 affects the function of AML cells, we performed the 3-(4,5)-dimethylthiahiazo (-z-y1)-3,5-di-phenytetrazoliumromide (MTT) analysis and found that knockdown of SAP30 significantly decreased the proliferation of AML cells (Supplementary information, Fig. S6m), and the q-PCR and Western blotting analysis revealed that knocking down SAP30 significantly increased the expression of MXD4 in human and murine AML cells (Fig. 6i, j; Supplementary information, Fig. S5i). To investigate the target genes of SAP30 in AML cells, we performed the CUT&Tag analysis by using the anti-SAP30 antibody in AML cells and found that SAP30 directly bound to *MXD4* promoter (Fig. 6k). The profile and heat-map of signal peaks showed that the binding sites of SAP30 on genes are mainly on the TSS (Supplementary information, Fig. S6n), distal intergenic regions, intron and promoters (Supplementary information, Fig. S6o). Meanwhile, the ChIP assay showed that UHRF1 is less enriched on *MXD4* promoter after SAP30 knockdown in AML cells (Fig. 6l). The expression of UHRF1 but not UHRF1-Mut2 mutant rescued UHRF1 suppression-induced *MXD4* upregulation at both mRNA and protein levels in AML cells (Fig. 6m; Supplementary information, Fig. S6p). The enrichment of UHRF1 on *MXD4* promoter is significantly decreased in Mut2-expressing THP-1 cells with UHRF1 knockdown compared with UHRF1-expressing AML cells (Supplementary information, Fig. S6q). The DNA methylation analysis showed that expression of UHRF1-Mut2 mutant did not rescue UHRF1 suppression-induced decrease of DNA methylation level of *MXD4* in AML cells (Fig. 6n, o). Taken together, UHRF1 interacts with SAP30 through G572 and F573, and this interaction plays a vital role in MXD4 repression and leukemogenesis.

### A newly discovered UF146 suppresses the survival of AML cells in vitro

**Fig. 7:**
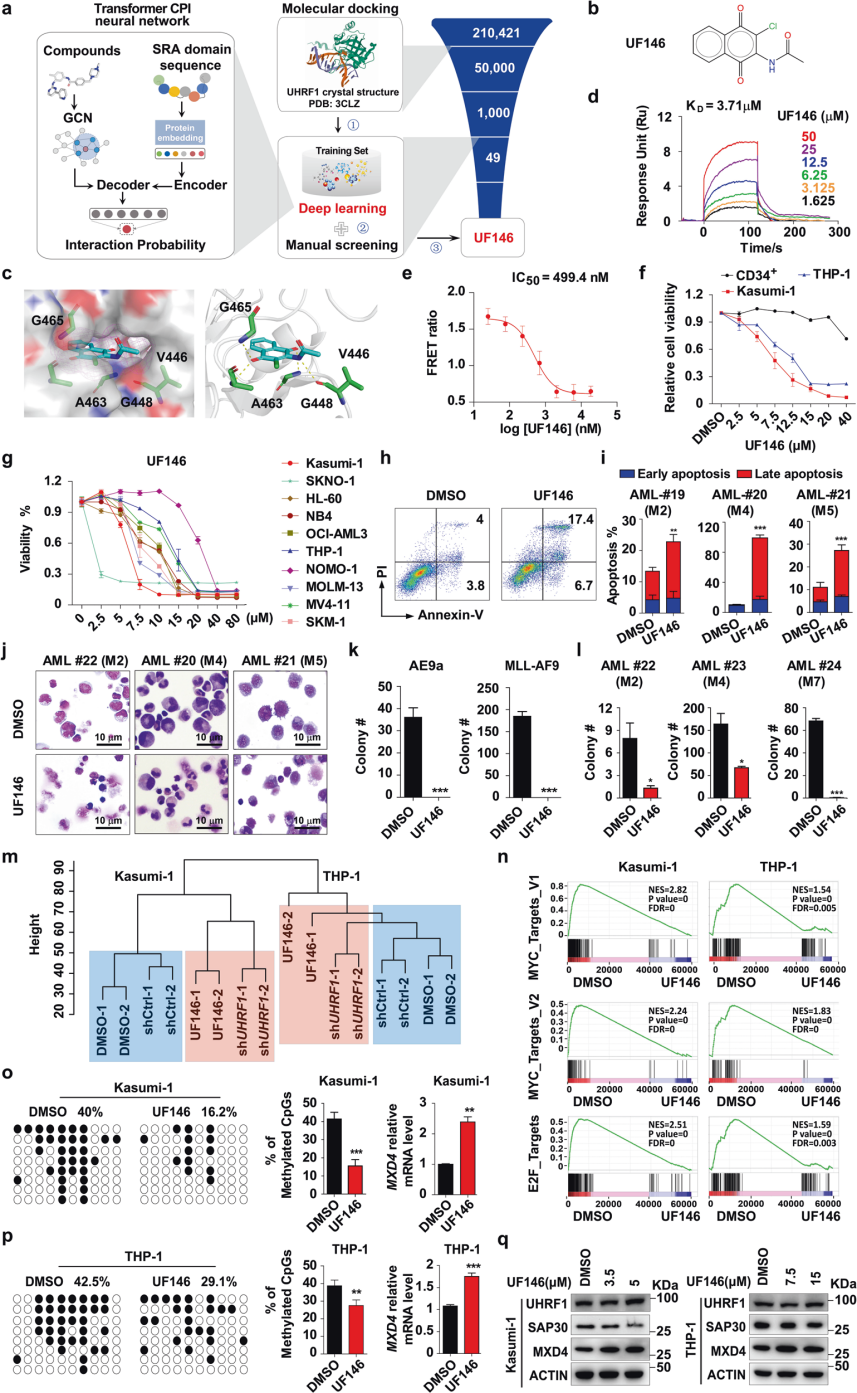
UHRF1 inhibitor UF146 suppresses AML cell survival by inhibiting proliferation and promoting apoptosis in vitro. **a** The scheme of the screening protocol for UHRF1 inhibitor. **b** The structural formula of UHRF1 inhibitor UF146. **c** The binding mode of UF146 to the G465, A463, G448 and V446-created 5mC cavity in SRA domain. **d**, **e** The SPR (**d**) and FRET (**e**) analysis were performed to examine the direct binding affinities of UF146 to the SRA domain of UHRF1 (*n* = 3). **f** The cellular viability of human CD34^+^ HSPCs, Kasumi-1 and THP-1 cells treated with UF146 or the vehicle was examined by MTT assay (*n* = 3). **g** The cellular viability of AML cells (Kasumi-1, SKNO-1, HL60, NB4, OCI-AML3, THP-1, NOMO-1, MOLM-13, MV4-11 and SKM-1 cells) treated with UF146 or the vehicle control for 48 h. **h**, **i** The representative flow cytometry analysis profiles (**h**) and quantification (**i**) analysis of the early apoptosis and late apoptosis in the primary AML patient cells 24 h after the treatment of UF146 or the vehicle control (*n* = 3). **j** The Wright’s staining of the primary AML cells treated with the vehicle or UF146. **k** The number of colonies generated from AE9a- or MLL-AF9-driven LICs treated with UF146 or the vehicle (*n* = 3). **l** The number of colonies generated from the primary AML patient cells treated with UF146 or the vehicle (*n* = 3). **m** The cluster dendrogram analysis of AML cells treated with UF146 and AML cells with knockdown of UHRF1. **n** The RNA-seq and GSEA analysis of the AML cells treated with UF146 or the vehicle. **o**, **p** The DNA methylation levels of *MXD4* in UF146- or the vehicle-treated Kasumi-1 (**o**) and THP-1 (**p**) cells were analyzed by the bisulfite sequencing (*n* = 3). **q** Western blotting analysis of UHRF1/MXD4/SAP30 in AML cells 24 h after UF146 treatment. Statistical analyses were performed using Student’s unpaired *t*-test for **i**, **k**, **l**, **o** and **p**. *P < 0.05, **P < 0.01, ***P < 0.001.

To find a specific UHRF1 inhibitor, we performed a screening using structure-based molecular docking and sequence-based deep learning.^44^ The crystal structure of UHRF1 (PDB accession code: 3CLZ) was selected for molecular docking via the Maestro module of Schrödinger software package.^14^ A total of 49 candidate compounds were finally experimentally evaluated (Fig. 7a, b). The docking pose showed that UF146 made hydrogen bonds with the SRA domain groove of UHRF1 through G465, V446, A463 and G448 (Fig. 7c). The surface plasmon resonance (SPR) analysis revealed that the binding of UF146 with the SRA domain exhibited a fast-on, fast-off kinetic pattern with a *K*_d_ of 3.71 µM (Fig. 7d). The fluorescence resonance energy transfer (FRET) analysis showed that UF146 significantly inhibits the binding of UHRF1 and hemi-methylated DNA with the IC_50_ of 499.4 nM (Fig. 7e).

Moreover, we examined the effect of UF146 on the viability of Kasumi-1, THP-1, NB4, SKM-1, HL-60, OCI-AML3, MOLM13, NOMO-1, MV4-11 cells or human CD34^+^ hematopoietic stem and progenitor cells (HSPCs) isolated from cord blood. The MTT analysis showed that UF146 significantly inhibited the growth of AML cells with minimal effect on proliferation of normal HSPCs and their DNA methylation state (Fig. 7f, g; Supplementary information, Fig. S7e). The currently available UHRF1 inhibitors NSC232003, Proanthocyanidins and Baicalein had no effect on AML cells (Supplementary information, Fig. S7c, d). The UF146 treatment significantly increases the apoptosis and induces the cell cycle arrest at G2/M phase in AML cells (Supplementary information, Fig. S8a‒d). UF146 also induces the apoptosis of primary leukemia cells isolated from AML patients (Fig. 7h‒j), and strikingly inhibits the expansion of primary AML cells and AE9a/MLL-AF9-expressing LICs in the CFU assays (Fig. 7k, l).

To investigate how UF146 inhibits the growth of AML cells, we performed the comparative RNA-seq analysis of Kasumi-1 and THP-1 cells with UF146 treatment. The results revealed that 932 genes were significantly upregulated, and 276 genes were downregulated in both Kasumi-1 and THP-1 cells (Supplementary information, Fig. S8f). The GO analysis of DEGs showed that GO terms related to myeloid cell homeostasis, epigenetic gene expression regulation pathways, DNA binding transcription factor binding, cell cycle-related and apoptotic signaling pathways were highly enriched (Supplementary information, Fig. S8g, h). Furthermore, we applied the GSEA analysis to identify the pathways regulated by UF146 in AML cells and found the GSEA hallmark gene sets of the MYC targets, E2F targets, G2/M checkpoint and apoptosis signaling pathways were among top enriched gene sets that are regulated by UF146 (Fig. 7n; Supplementary information, Fig. S8i), which is consistent with the analysis about the pathways regulated by UHRF1. The cluster analysis of RNA-seq data showed that AML cells with UHRF1 knockdown and UF146-treated AML cells can be grouped together and are separated from the control groups (Fig. 7m). A significant overlap of DEGs (Supplementary information, Fig. S7f) caused by UHRF1 knockdown and UF146 treatment of AML cells was observed, indicating that UF146 is a specific inhibitor of UHRF1. Moreover, the bisulfite sequencing results revealed that the DNA methylation levels of CpG sites around the *MXD4* transcription start sites are decreased in UF146-treated AML cells compared with the control cells (Fig. 7o, p). UF146 treatment impaired the enrichment of UHRF1 on *MXD4* promoter and upregulated *MXD4* expression without affecting the expression of UHRF1 (Fig. 7q; Supplementary information, Fig. S7g, h). Taken together, UF146 treatment leads to MXD4 activation and the downregulation of its downstream MYC and E2F pathways by targeting UHRF1, resulting in the inhibition of leukemic potential of AML cells.

### UF146 has therapeutic efficacy on AML in vivo

**Fig. 8:**
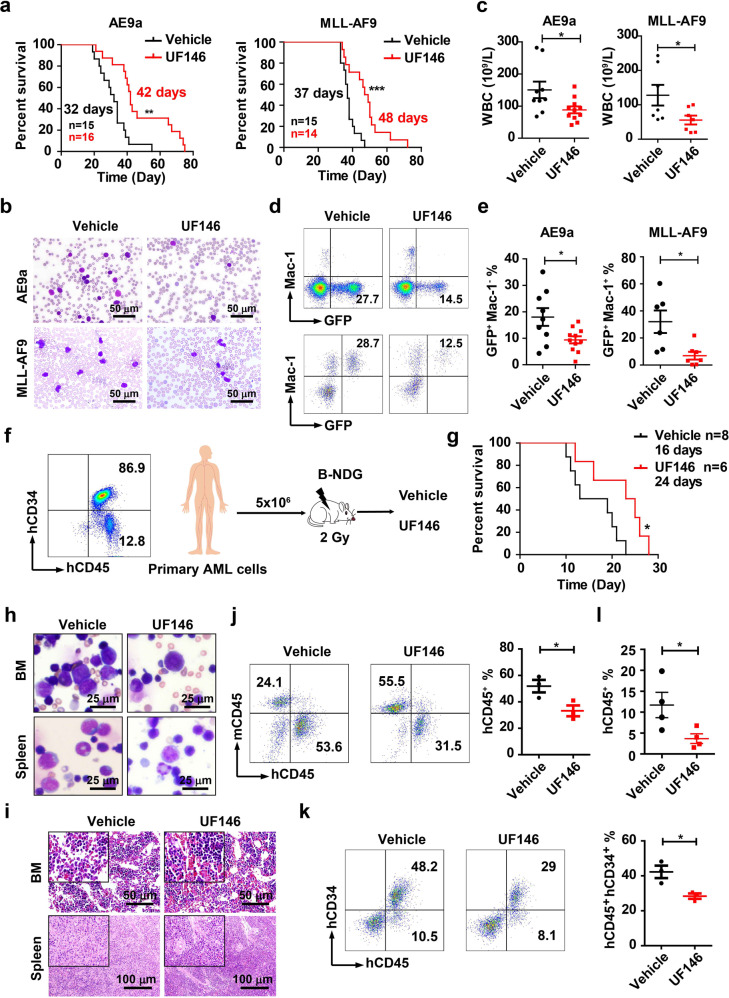
UF146 prolongs the survival of AML mice. **a** The survival of AE9a- (*n* ≥ 15) or MLL-AF9-driven (*n* ≥ 14) AML mice treated with UF146 or the vehicle. **b** The Wright’s staining of PB isolated from the AE9a (at week 3)- or MLL-AF9 (at week 4)-driven AML mice treated with UF146 or the vehicle. **c** The WBC counts in the AE9a- or MLL-AF9-driven AML mice treated with UF146 or the vehicle (*n* ≥ 7). **d**, **e** The representative flow cytometry analysis profiles (**d**) and quantification analysis (**e**) of GFP^+^ Mac-1^+^ PB cells of UF146- or the vehicle-treated mice (*n* ≥ 8). **f** The strategy of the AML patient cell-derived xenograft transplantation model. **g** The survival curves of the UF146- or the vehicle-treated B-NDG mice (*n* ≥ 6) transplanted with the primary AML-M4 cells. **h**, **i** The Wright’s (**h**) and HE (**i**) staining analysis of BM and spleen cells isolated from UF146- or the vehicle-treated recipients that were transplanted with the primary AML-M4 cells were performed 2 weeks after transplantation. **j**, **k** The flow cytometry analysis of human CD45^+^ (hCD45^+^) (**j**), hCD45^+^human CD34^+^ (hCD34^+^) (**k**) BM cells isolated from UF146- or the vehicle-treated recipients transplanted with the primary AML-M4 cells (*n* = 3). **l** Flow cytometry analysis of hCD45^+^ PB cells from UF146- or vehicle-treated recipient mice transplanted with the primary AML-M2 cells (*n* = 4). Data are all presented as means ± SD; *P < 0.05, **P < 0.01, ***P < 0.001.

To investigate the therapeutic effect of UF146 on AML in vivo, we transplanted AE9a- or MLL-AF9-expressing leukemic cells into the sublethally irradiated recipient mice. UF146 (2.5 mg/kg, intraperitoneal injection, every other day for three weeks) treatment started three days after the transplantation. UF146 significantly delayed the leukemia onset and led to significant increases in the survival time, extending the median OS from 32 to 42 days (AE9a-driven leukemia model) or 37 to 48 days (MLL-AF9-driven leukemia model) without the body weight loss (Fig. 8a; Supplementary information, Fig. S9a, b). Three weeks after the transplantation, the morphology analysis of PB indicated that the infiltrated leukemic cells were decreased in UF146-treated recipients compared with the control recipients (Fig. 8b). In addition, the complete blood count (CBC) analysis revealed fewer WBCs in PB of UF146-treated recipients (Fig. 8c), with far fewer GFP^+^ leukemia cells (Fig. 8d, e).

To further confirm that the specific effect of UF146 on AML cells is specifically due to the inhibition of UHRF1, we performed the CFU assay and found that UF146 significantly inhibits the colony formation of the control AML cells with little effect on AML cells with UHRF1 knockdown (Supplementary information, Fig. S8e). Western blotting analysis showed that the cleavage of PARP, the apoptosis-related protein, was significantly increased in UF146-treated THP-1 control cells compared with UF146-treated THP-1 cells with UHRF1 knockdown (Supplementary information, Fig. S7i). Moreover, knocking down MXD4 partially rescued the phenotype of UF146 treatment such as apoptosis and cell cycle arrest in AML cells (Supplementary information, Fig. S7j‒n). We further transplanted MLL-AF9*Uhrf1*^*fl/fl*^ or MLL-AF9*Uhrf1*^Δ/Δ^ leukemic cells into the sublethally irradiated recipient mice and treated these mice with UF146 as described above. We found that UF146 significantly delayed the leukemia development in the *Uhrf1*^*fl/fl*^ group but not in the *Uhrf1*^Δ/Δ^ group (Supplementary information, Fig. S9c, d), indicating that UF146 specifically targets Uhrf1 in vivo.

Our data have shown that UHRF1 knockdown or UF146 treatment significantly inhibited the proliferation of OCI-AML3 cells, suggesting UHRF1 inhibition could be effective in treating M4 subtype AML. We next evaluated the effects of UF146 on the AML PDX model, which we happened to have. We transplanted 5 × 10^6^ BM mononuclear cells from AML-M2 or M4 patients to the sublethally irradiated B-NDG mice, and 3 days after transplantation, we treated the recipients with UF146 (2.5 mg/kg, intraperitoneal injection, every other day) (Fig. 8f). Two weeks after the transplantation, the flow cytometry analysis of BM (M4) or PB (M2) cells showed fewer hCD45^+^ or hCD45^+^CD34^+^ leukemia cells in the UF146 treatment group (Fig. 8j‒l). The morphology and histology analysis of BM and spleen indicated that the infiltrated human leukemic cells (M4) were highly decreased in UF146-treated recipients, compared with the control group (Fig. 8h, i). UF146 also significantly prolonged the median survival (M4) of the recipients, compared with the control mice (Fig. 8g). Above all, these data demonstrated the therapeutic potential of UF146 in AML.

To evaluate the effects of UF146 on normal hematopoiesis, we treated wild type mice with 2.5 mg/kg UF146 for three weeks. The body weight, total BM number and PB counts of WT mice were not changed by UF146 treatment (Supplementary information, Fig. S9e‒g), and the size and weight of spleen or liver were also unchanged (Supplementary information, Fig. S9i, j). The flow cytometry analysis showed the normal frequencies of HSPCs and erythroid cells in BM (Supplementary information, Fig. S9k, l). The mature hematopoietic cells including lymphocytes and myeloid cells in BM, spleen or PB also displayed no significant differences in the mice treated with UF146 compared with the control mice (Supplementary information, Fig. S9m‒o). Thus, these results reveal that UF146 has no significant effects on normal hematopoiesis in mice.

## Discussion

AML is characterized by the increased self-renewal of HSPCs, and we have studied the relevance of UHRF1 in the self-renewal and development of the AML models. Our results show that UHRF1 is indispensable for self-renewal of LICs and loss of Uhrf1 delays leukemia development and initiation (Supplementary information, Fig. S9h). Limiting dilution assays demonstrate impaired LIC self-renewal, consistent with impaired in vivo cancer progression. Our results provide critical genetic evidence for an important role of UHRF1 in the development and progression of AML and suggest that targeting LIC self-renewal by pharmacologic inhibition of UHRF1 may be useful for AML treatment. Although our previous study showed that Uhrf1 deficiency led to decreased self-renewal of normal HSCs,^20^ Uhrf1 heterozygous deletion in mice did not change birth ratios,^20^ cell numbers, frequencies of HSPCs and multilineage differentiated blood cells in BM cells of mice (Supplementary information, Fig. S10a‒e). UHRF1 inhibitor UF146 significantly inhibited the self-renewal of LICs with minimal effect on human CD34^+^ HSPCs and normal hematopoiesis in wild type mice. Intriguingly, the effects of UF146 on LICs of AML and HSCs are different. Clearly, these divergent functions of UF146 between HSCs and LICs make UF146 an optional therapy for AML.

Suppressing UHRF1 triggered a unique gene signature in AML cells, including downregulation of the leukemia stem cell and MYC pathways. We also observed that UHRF1 inhibition induced inactivation of the MYC downstream target, E2F signaling, which was reported to contribute to the pathogenesis of myeloid malignancies.^45^ Consistent with our study, downregulating the MYC signature has been shown to decrease the self-renewal and proliferation of leukemic cells.^46,47^ It appears that decreased MYC signaling induced by UHRF1 inhibition confers a growth disadvantage to AML cells. We identified, *MXD4*, the MYC antagonist, as a target gene of UHRF1 in AML cells. MXD4 has been implicated in the mutated Flt signaling of Flt3-ITD-induced myeloproliferative disease, which indicates that MXD4 may play a critical role in myeloid malignancies.^48^ Our findings that blocking MXD4 weakens the effect of UHRF1 defects on self-renewal of LICs indicate that MXD4/MYC/E2F axis functioned as an important regulator of leukemogenesis.

The binding of SAP30, a component of histone deacetylase complex, to the gene promoter has been shown to result in the repression of transcription.^49–51^ SAP30 is essential for UHRF1-mediated repression of *MXD4* in AML cells. The G572 and F573 in the SRA domain of UHRF1 provide the docking sites for SAP30, allowing UHRF1 and SAP30 to colocalize at the regulatory regions of *MXD4* gene. which is involved in the regulation of LICs self-renewal. The important consequence of this interaction is that the promoter of *MXD4* is methylated, an event essential for the activation of MYC signaling pathway and the self-renewal-promoting effects. Thus, although SAP30 can bind to UHRF1 and presumably bring it to the gene promoter, the key step is the methylation of *MXD4* promoter, which perhaps contributes to leukemogensis at least in part by upregulating MYC target genes such as *E2F1* or *E2F2*. The discovery that UHRF1/SAP30-mediated methylation of *MXD4* promoter contributes potently to leukemogenesis suggests that inhibition of this methylation merits exploration as a possible therapeutic strategy for AML.

In the therapy of acute promyelocytic leukemia patients, the all trans retinoid acid/arsenic trioxide combination selectively targets the PML-RARα oncoprotein,^52^ and targeting the BCL-ABL tyrosine kinase has been the most effective therapy strategy for the majority of patients with chronic myelocytic leukemia.^53–55^ Although several novel drugs have been approved by FDA for AML patients since 2017,^56^ it is urgent to seek effective therapeutic targets for AML. As the critical roles of UHRF1 in the prognosis and pathogenesis of AML, it is prospective that UHRF1 can be a common therapeutic target for pan-AML. Some UHRF1 inhibitors have been developed^57–60^; however, some of them did not successfully target UHRF1 in AML cells or inhibit the growth of AML cells. Although the UHRF1 inhibitors Mitoxantrone and Hinokitiol inhibited the proliferation of AML cells, they also targeted USP11, PIM1 kinase, Snail or protein kinase B besides UHRF1 in cells.^61–64^ Although it has been reported that 4-BPC is the inhibitor of UHRF1, the chemical concentration of AML cell inhibition is much higher compared with UF146 (Supplementary information, Fig. S7c). Moreover, we found that 4-BPC inhibited the proliferation of human CD34^+^ cord blood cells (data not shown). In this study, we identified a UHRF1-specific inhibitor UF146 that is effective for AML cells and AML mouse models. Interestingly, UF146 disrupts the DNA methylation maintenance on *MXD4* promoter, resulting in the activation of *MXD4* in AML cells. Thus, targeting the DNA methylation function of UHRF1 by UF146 might be a promising strategy for AML therapy (Supplementary information, Fig. S10f).

## Materials and methods

### Cell lines

Kasumi-1, HL-60, NB4, OCI-AML3, THP-1, NOMO-1, MOLM-13, MV4-11, SKM-1 and AE9a cells were cultured in RMPI 1640 medium with 10% fetal bovine serum. MLL-AF9 cells were cultured in RMPI 1640 medium supplemented with 10 ng/mL SCF, 10 ng/mL IL-3 and 10 ng/mL IL-6. SKNO-1 cells were cultured in RMPI 1640 medium supplemented with 10 ng/mL GM-CSF. The reagent information are listed on the Supplementary information, Table S1.

### Patient samples, isolation and culture

AML patient samples were from Shanghai Children’s Medical Center, and the research was approved by the institution review board of the center (ER-SIBS-241904). Mononuclear cells were isolated from the primary BM cells with Ficoll (Norway), and cultured in RMPI 1640 medium with 20% fetal bovine serum. RNA-seq of AML patient cells was performed as described previously.^38^ The patients were stratified into groups based on their gene expression patterns, and the Kaplan-Meier method and log-rank test were used to analyze the relapse rate of different groups of the patients.

### Mice

The *Uhrf1*^*fl*/*fl*^ mice were obtained as described previously.^20^ All mice were bred and housed in specific pathogen-free conditions. *Uhrf1*^Δ/Δ^ mice were generated by crossing *Uhrf1*^*fl/fl*^ mice with *Mx1*-Cre and Cre-ER mice. *Uhrf1* deletion in BM or fetal liver cells from *Uhrf1*^*fl/fl*^ and *Uhrf1*^*fl/fl*^ *Mx1*-Cre mice was induced by poly(I:C) (3 intraperitoneal injections at a dosage of 10 mg/kg every other day were given). *Uhrf1* deletion in BM or fetal liver cells from *Uhrf1*^*fl/fl*^ and *Uhrf1*^*fl/fl*^ Cre-ER mice was induced by 4-OH Tamoxifen in vitro at the centration of 1 µM. All mice were genotyped by PCR (primers for genotyping are listed in Supplementary Table S2). All animal experiments were approved by the Institutional Animal Care and Use Committee of the Shanghai Institutes for Biological Sciences, Chinese Academy of Sciences.

### RNA extraction and quantitative RT-PCR

Total RNA was extracted with the TRIzol Reagent (Invitrogen), then reversely transcribed to cDNA by using the PrimeScript RT reagent Kit (TaKaRa). Real-time PCR was performed by using the SYBR Green Supermix (Roche) on the machine ABI Q7. The pair primers are listed on Supplementary information, Table S2.

### Purification and culture of cord blood-derived CD34^+^ cells

Human cord blood samples were from Shanghai Children’s Medical Center. Mononuclear cells were isolated from cord blood samples with Ficoll (Norway), and CD34^+^ cells were purified by the human CD34 MicroBead Kit (MACS, Miltenyi Biotec). Purified CD34^+^ cells were then cultured in the IMDM medium containing BIT9500 (Stem Cell technologies), 100 ng/mL SCF, 100 ng/mL FLT3, 100 ng/mL TPO and 20 ng/mL IL-6. The reagent information are listed on Supplementary information, Table S1.

### Immunohistochemistry

AML patient BM paraffin section samples were dewaxed and rehydrated by xylene 3 times, absolute ethanol twice, 95% ethanol, 85% ethanol and 75% ethanol, and then subjected to antigen retrieval by citric acid repair solution for 10 min. 3% H_2_O_2_ was used to eliminate endogenous peroxidase and 1% BSA was used to block the samples. Samples were stained with the anti-UHRF1 antibody (1:1500; Bioworld Technology, BS7227) overnight at 4 °C, and then subjected to secondary antibody and color development with DAB (Dako, K5007) for 3 min. Samples were stained with hematoxylin for 5 min, and then dehydrated by 75% ethanol, 85% ethanol, 95% ethanol once, absolute ethanol twice and xylene three times. Slides were sealed with neutral resin, and then analyzed on the microscope.

### Transplantation assay

E14.5 fetal liver cells were isolated from *Uhrf1*^*fl/fl*^ or *Uhrf1*^*fl/fl*^ *Mx1*-Cre mice and cultured in in RMPI 1640 medium supplemented with 10 ng/mL SCF, 10 ng/mL IL-3 and 10 ng/mL IL-6. These cells were infected with retrovirus expressing AE9a using 8 µg/mL polybrene (Merck/millipore). Approximately 1 × 10^5^ GFP^+^ fetal liver cell mixed with 5 × 10^5^ helper cells were injected into the tail vein of lethally irradiated (9.5 Gy) recipient mice. For BM transplantation, we isolated BM cells from 5-FU-treated *Uhrf1*^*fl/fl*^ or *Uhrf1*^*fl/fl*^ *Mx1*-Cre mice and cultured them in the medium. These BM cells were infected with retrovirus expressing MLL-AF9. 1 × 10^4^ GFP^+^ BM cell mixed with 5 × 10^5^ helper cells were injected into the tail vein of lethally irradiated (9.5 Gy) recipient mice. To knock down *Uhrf1* in vivo, we transduced AE9a or MLL-AF9 leukemia cells with shRNA against *Uhrf1* and treated cells with puromycin for 48 h. 1 × 10^4^ GFP^+^ cells were injected into the tail vein of sublethally irradiated (4.75 Gy) recipient mice. Survival of the mice was monitored daily.

### Hematoxylin-eosin (HE) staining

Mouse tissues were sequentially placed in 75% alcohol, 85% alcohol, 95% alcohol and pure alcohol for gradient dehydration, transparency, paraffin infiltration and embedding, and sectioning was performed. For HE staining, the dried sections were dewaxed and rehydrated by xylene 3 times, absolute ethanol twice, 95% ethanol, 85% ethanol and 75% ethanol once. After hematoxylin stained, sections were washed and treated with 1% alcohol hydrochloride, and then stained with 1% ammonia aqueous solution. Eosin was used to stain the cytoplasm, and xylene was used to transparentize the sections. Finally, the sections were fixed with neutral resin for microscope imaging.

### Cytospin and Wright’s staining

Murine PB cells were smeared onto glass slides, and other AML cells were fixed onto glass slides by cytospin (Cytopro, AC-160). We added about 0.5 mL–0.8 mL Wright’s solution A (Jiancheng Biotech, D007) on the slides, and let the dye solution cover the entire specimen for 1 min. We then added Wright’s solution B (Jiancheng Biotech, D007) on the slides (3 times volume of solution A), mixed the two liquids thoroughly, and incubated them for 3–10 min. After Washing and drying, cell morphology was analyzed by microscope imaging.

### Colony formation assay and replating

For colony assay of AML cells (Kasumi-1, THP-1, AE9a and MLL-AF9 cells), cells were infected with lentivirus expressing shRNA against UHRF1 for 48 h (scrambled shRNA as control) using PLKO.1 vector. After AML cells were selected with puromycin for 48 h, they were cultured in Methocult GF-H4435 or Methocult GF-M3434 medium (Stem Cell Technologies). *Uhrf1*^*fl/fl*^ Cre-ER/*Uhrf1*^*fl/fl*^ fetal liver cells were isolated from E14.5 embryos, and *Uhrf1*^*fl/fl*^ Cre-ER/*Uhrf1*^*fl/fl*^ BM cells were isolated from 6‒8 weeks old mice. These cells were cultured in the RMPI 1640 medium with 10% fetal bovine serum supplemented with 100 ng/mL SCF, 10 ng/mL IL-3 and 10 ng/mL IL-6, and were infected with MIGR1-based retrovirus expressing AML1-ETO-9a (AE9a) or MLL-AF9 (MA9). GFP^+^LSK (fetal liver) or GFP^+^L-GMP (BM) cells were sorted 48 h after the infection. These LICs were cultured in Methocult GF-M3434 medium (Stem Cell Technologies) supplemented with 1 µM 4-OH-Tamoxifen. The colonic cells were replated weekly. All colony number was counted on day 7 using an inverted system microscope (Olympus).

### CAFC assay

4000 *Uhrf1*^*fl/fl*^ Cre-ER and *Uhrf1*^*fl/fl*^ E14.5 fetal liver LSK cells expressing AE9a or 500 BM L-GMP cells expressing MLL-AF9 were seeded onto MS5 stroma cells respectively and cultured in α-MEM containing 12.5% FBS, 12.5% horse serum, 1 μM hydrocortisone and 1 mM glutamine supplemented with 1 µM 4-OH-Tamoxifen. Medium was semi-replenished every week, and “cobblestone” colonies were scored at week 5. CAFC frequency was determined using L-Calc software (Stem Cell Technologies).

### Apoptosis, cell proliferation and cell cycle assay

AML cells were stained with Annexin-V and Propidiumiodide (PI) purchased from BD Biosciences and Cell Signaling Technology. Cell viability was determined by MTT (Meilunbio) and lysis solution (10% SDS, 5% isobutanol and 0.012 M HCL). Cells were fixed by 75% ice ethanol over 24 h before cell cycle assay, and then were stained with PI.

### RNA sequencing

AML (Kasumi-1 and THP-1) cells were infected with lentivirus expressing shRNA against *UHRF1* for 48 h (scrambled shRNA as control). Then AML cells were selected with puromycin for 48 h, and RNA was extracted with the TRIzol Reagent (Invitrogen). RNA-seq library was carried out using the TruSeq RNA Sample Preparation Kit v2 (Illumina) and sequenced with Illumina HiSeq2500. TopHat (version 2.0.9) was used to align the reads to the genome and HT-seq was used to calculate FPKM (fragments per kilobase of exon model per Million mapped reads) of the genes. GSEA and GO analyses were performed to analyze the differentially expressed genes.

### CUT&Tag sequencing

CUT&Tag assay was performed using NovoNGS CUT&Tag 3.0 High-Sensitivity Kit (for Illumina, Novoprotein scientific Inc., Cat#N259-YH01-01A). Briefly, 1 × 10^5^ Kasumi-1 or THP-1 cells were harvested freshly. Then 37% formaldehyde was gently added to cells (the final concentration of formaldehyde is 0.1%) and these samples were incubated at room temperature for 2 min. Cross-linking was stopped by addition of 1.25 M glycine and then the samples were washed with the wash buffer. The cells were enriched by ConA Beads and resuspended by 50 µL primary antibody buffer of anti-UHRF1 antibody (1:100, Bioworld Technology, BS7227) or anti-SAP30 (1:100; Abcam, ab231804) antibody, and incubated overnight at 4 °C. We then discarded the primary antibody buffer and added 100 µL anti-rabbit IgG antibody buffer for 1 h at a dilution of 1:100. Then the beads were washed for 3 times using antibody buffer and then incubated with proteinA/G-Tn5 transposome for 1 h and washed for 3 times by ChiTaq buffer. Cells were resuspended in 50 µL Tagmentation buffer (10 mM MgCl_2_ in ChiTaq Buffer) and incubated at 37 °C for 1 h. The incubation was stopped by adding 10 µL 10% SDS at 55 °C for 10 min. The DNA fragments were extracted by Tagment DNA extract beads and amplified using 5× AmpliMix. Then the DNA was re-extracted by DNA clean beads for sequencing.

### Bisulfite sequencing

The genomic DNA of Kasumi-1 and THP-1 cells was extracted (Tiangen, DP304-02), and 500 ng genomic DNA was used to CT conversion (Zymo Research, D5020). The target fragment of *MXD4* was amplified by PCR (Forward-TAGTAGTTAGGAGGGTGGAAATTTT, Reverse-CCACCAATATAAAAATCCTCTTTTT), and obtained by running and recycling agarose gel (Tiangen, DP209-03). The target fragment was ligated with PMD8-T vector (Takara, 6011) for 2 h at 16 °C. To get the monoclonal strains, we transduced the ligation products into *E. coli* competent cell. The level of methylation was sequenced and analyzed.

### GST pull-down assay

BL21 *E.coli* cells were transduced with the plasmids for GST-UHRF1-UBL, GST-UHRF1-TPD, GST-UHRF1-SRA, GST-UHRF1-RING, GST-UHRF1 full length and His-SAP30, and then induced by isopropyl-β-d-thiogalactoside (1 mM) at 16 °C for 8 h. Cells were collected and suspended in lysis buffer (50 mM Tris-HCL, pH 7.5, 100 mM NaCl, 1% Triton, 1 mM PMSF) supplemented with protease-inhibitor cocktail (Biomake) and sonicated (10 cycles of 10 s each at 30% output). The supernatant after sonication and centrifugation was incubated with anti-GST antibody-conjugated beads to enrich recombinant proteins. The immunoprecipitate was washed with lysis buffer before incubation with His-SAP30 protein overnight at 4 °C. The beads were collected by centrifugation and washed six times with lysis buffer before the immunoblotting analysis.

### Co-IP

For the endogenous IP assay, Kasumi-1 and THP-1 cells were collected and resuspended with T/G buffer (20 mM Tris-HCI, pH 7.5, 300 mM NaCl, 2 mM EDTA, 1% Triton X-100, 20% Glycerol and 1 mM PMSF) on ice for 1 h. The antibodies and magnetic beads were sequentially added into the supernatant for the subsequent experiments. For the exogenous HA-IP assay, PLVX-GFP-UHRF1 truncated plasmid and PLVX-puro-SAP30 plasmid were simultaneously transfected into 293T cells by Polyethylenimine Linear (PEI) transfection reagent. The cells were collected 48 h after transfection and lysed with IP lysis buffer (20 mM HEPES, pH 7.4, 150 mM NaCl, 1 mM EDTA, 0.25% Sodium deoxycholate, 0.5% NP-40, and 1 mM PMSF). The supernatant after lysis was incubated with anti-HA antibody-conjugated beads to enrich recombinant proteins overnight at 4 °C. The beads were collected by centrifugation and washed three times with IP lysis buffer before the analysis of immunoblotting.

### Screening of small molecule inhibitors of UHRF1

To find UHRF1 inhibitors, a virtual screening of chemical library was performed. Firstly, the prepared Specs Library (210,421 compounds) and UHRF1 (PDB: 3CLZ) docking was performed using Schrödinger software package (Schrödinger, LLC: New York, NY, 2017) in the high-throughput virtual screening (HTVS) mode. After ranking the compounds according to their docking score, the top 50,000 compounds were subject to molecular docking by Glide with standard precision mode. Secondly, to reduce the false-positive rate of docking-based virtual screening, a deep learning model based on TransformerCPI was also employed for hit selection. The top 1000 molecules were subject to further evaluation, and the candidate active molecules screened by the deep learning model and the molecules of the docking poses were merged, clustered, and selected manually. Finally, a total of 49 candidate compounds were purchased and then experimentally evaluated. The TransformerCPI score and docking score of the final 49 candidate compounds were shown in Supplementary information, Table S3.

### SPR

Biacore T200 instrument (GE Healthcare) was used to perform the SPR binding assays. The UHRF1-SRA domain protein was covalently immobilized onto a CM5 sensor chip in 10 mM sodium acetate, pH 4.5. Compounds were serially diluted and injected onto a sensor chip at a flow rate of 30 μL/min for 120 s (contact phase), followed by 120 s of buffer flow (dissociation phase). The equilibrium dissociation constant (*K*_d_) value was derived using Biacore T200 Evaluation software Version 1.0.

### FRET

Biotin-labeled hmDNA (biotin-GGGCCXGCAGGG) and his-SRA protein were used in FRET assay. In brief, diluted UF146 compound was preincubated with 50 nM his-SRA protein for 30 min in room temperature. After incubating, 40 nM Biotin-labeled hmDNA was added into mix. Followed by incubation for another 15 min, 10 μL beads (5 μL streptavidin-XL665 and 5 μL Anti His-Tb cryptate) were added into each well and incubated for 30 min. The FRET signals were detected using a Tecan Spark microplate reader (excitation at 620 nm and emission at 665 nm).

### PDX and CDX transplantation

Primary AML cells were isolated from BM of AML patients by using Lymphoprep (Stem cell, 07861), and 5 × 10^6^ primary AML cells were injected into the tail vein of sublethally irradiated (2.0 Gy) B-NDG recipient mice (NOD-*Prkdc*^*scid*^*IL2rg*^*tm1/*^Bcgen, BIOCYTOGEN, B-CM-002) for PDX transplantation. These recipient mice were treated with UF146 (2.5 mg/kg/day) or vehicle via intraperitoneal injection every other day 3 days after transplantation. The survival curve and AML cells infiltration assay were analyzed respectively. For CDX transplantation, indicated AML cells (3 × 10^6^ Kasumi-1 cells or 1 × 10^6^ THP-1 cells) were injected into the tail vein of sub-lethally irradiated (2.0 Gy) B-NDG recipient mice. The survival curve of recipient mice was analyzed.

## Quantification and statistical analysis

All data were expressed as the means ± SD of the mean. Results were obtained from 3 independent experiments. Statistical analyses were performed by using the GraphPad Prism with unpaird two-tailed Student’s *t*-test. The Kaplan-Meier method was used for survival curves, which were compared by the log-rank test. *P < 0.05; **P < 0.01; ***P < 0.001.

## Supplementary information

Supplementary information Fig 1

Supplementary information Fig 2

Supplementary information Fig 3

Supplementary information Fig 4

Supplementary information Fig 5

Supplementary information Fig 6

Supplementary information Fig 7

Supplementary information Fig 8

Supplementary information Fig 9

Supplementary information Fig 10

Supplementary Table S1

Supplementary Table S2

Supplementary Table S3

## Data Availability

RNA-sequencing and CUT&Tag Sequencing data sets generated in this study have been deposited in Gene Expression Omnibus database using the accession number GSE181519.
