# Supplementary material for: Targeting UHRF1-SAP30-MXD4 axis for leukemia initiating cell eradication in myeloid leukemia
Source: Cell Res. 2022 Oct 27;32(12):1105–23. doi: 10.1038/s41422-022-00735-6 (PMC9715639; doi:10.1038/s41422-022-00735-6)
Supplement: Supplementary file 1 — Supplementary information Fig 1 [file 41422_2022_735_MOESM1_ESM.pdf]

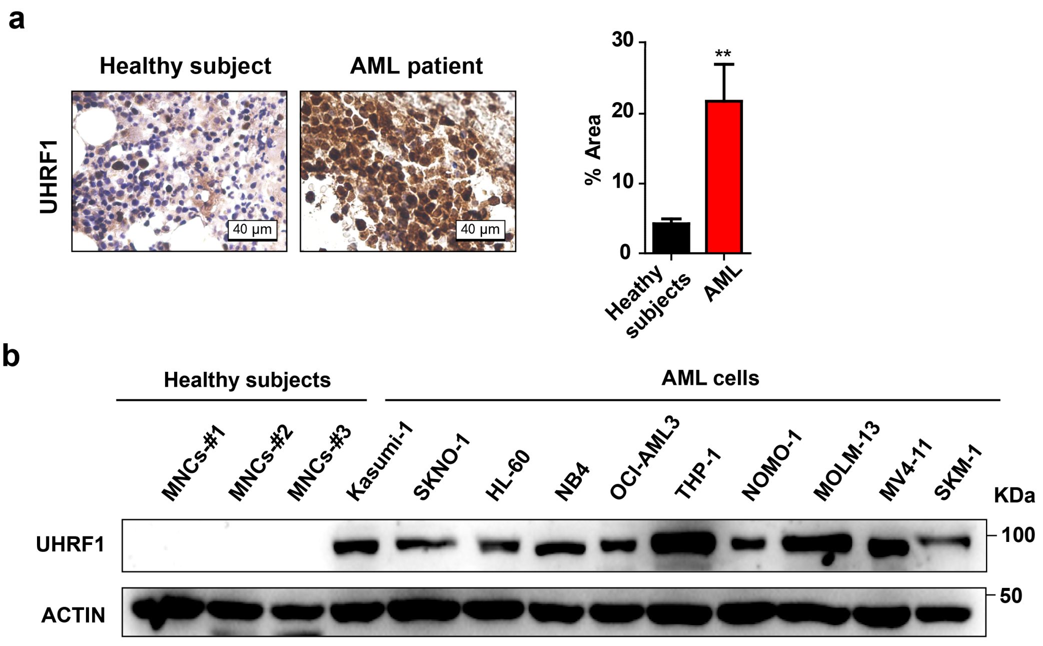

Supplementary information Fig. S1 UHRF1 is highly expressed in human AML cells.

a The immunohistochemistry analysis showed that UHRF1 expression is higher in BM of AML patients (n=4) compared with the healthy subjects (n=2). % Area means the percentage of area that can be positively stained by the anti-UHRF1 antibody. **b** The expression of UHRF1 was examined by Western blotting analysis for AML cell lines and healthy human MNCs. Statistical analyses were performed using student's unpaired t-test for **a**. *$P < 0.05$, **$P < 0.01$, ***$P < 0.001$.
