# Supplementary material for: Targeting UHRF1-SAP30-MXD4 axis for leukemia initiating cell eradication in myeloid leukemia
Source: Cell Res. 2022 Oct 27;32(12):1105–23. doi: 10.1038/s41422-022-00735-6 (PMC9715639; doi:10.1038/s41422-022-00735-6)
Supplement: Supplementary file 2 — Supplementary information Fig 2 [file 41422_2022_735_MOESM2_ESM.pdf]

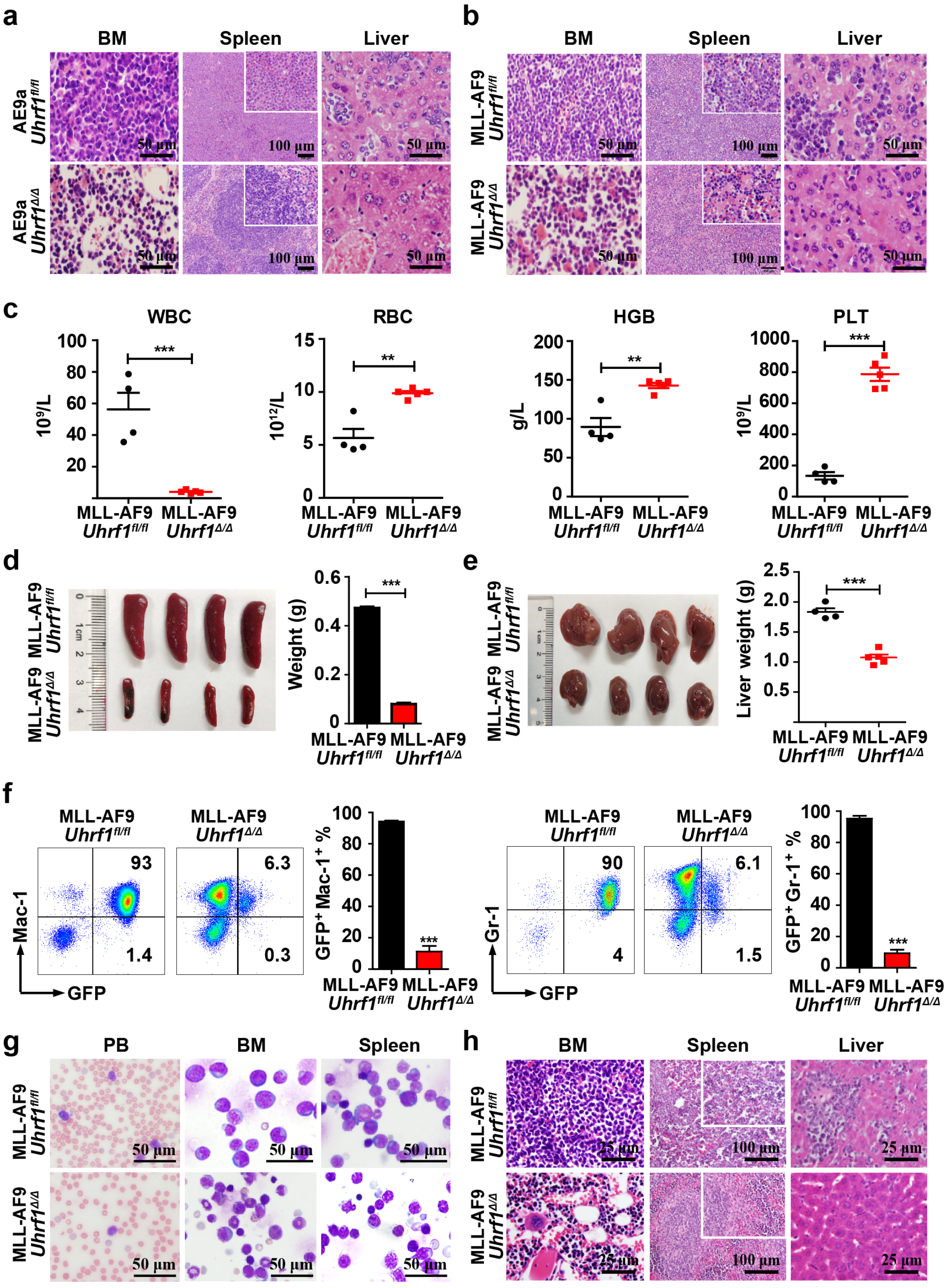

Supplementary information Fig. S2 Deletion of *Uhrf1* abrogates the progression of AML in mouse models.

a-b The BM, spleen and liver sections, stained with HE, of AE9a*Uhrf1*$^{fl/fl}$ /AE9a*Uhrf1*$^{\Delta/\Delta}$ (3 months after transplantation) (a) and MLL-AF9*Uhrf1*$^{fl/fl}$ /MLL-AF9*Uhrf1*$^{\Delta/\Delta}$ mice (6 weeks after transplantation) (b), were shown (scale bars of

BM and liver samples: 50 μM; scale bar of spleen samples: 100 μM). **c** The WBC counts of MLL-AF9*Uhrf1*$^{\Delta/\Delta}$ mice were significantly lower than MLL-AF9*Uhrf1*$^{fl/fl}$ mice 2 weeks after the secondary transplantation, while the RBC (red blood cells), PLT (platelets) and HGB (Hemoglobin) of MLL-AF9*Uhrf1*$^{\Delta/\Delta}$ were higher (n=4). **d-e** The size and weight of the spleen (**d**) and liver (**e**) of MLL-AF9*Uhrf1*$^{\Delta/\Delta}$ mice were decreased compared with MLL-AF9*Uhrf1*$^{fl/fl}$ mice 2 weeks after the secondary transplantation (n=4). **f** The percentages of GFP^{+}Mac-1^{+} and GFP^{+}Gr-1^{+} cells in the BM of MLL-AF9*Uhrf1*$^{\Delta/\Delta}$ mice were significantly less than those of MLL-AF9*Uhrf1*$^{f/f}$ mice 2 weeks after the secondary transplantation (n=4). **g** The morphology analysis showed that PB, BM and spleen had less leukemia blast cells in the MLL-AF9*Uhrf1*$^{\Delta/\Delta}$ group compared with MLL-AF9*Uhrf1*$^{fl/fl}$ group 2 weeks after the secondary transplantation (scale bar: 50 μM). **h** The BM, spleen and liver sections of MLL-AF9*Uhrf1*$^{fl/fl}$ and MLL-AF9*Uhrf1*$^{\Delta/\Delta}$ mice, stained with HE, are shown 2 weeks after the secondary transplantation (scale bars of BM and liver samples: 25 μM; scale bar of spleen samples: 100 μM). Data are all presented as mean ± SD. Statistical analyses were performed using student's unpaired t-test for **c**, **d**, **e** and **f**. *p<0.05, **p<0.01, ***p<0.001.
