# Supplementary material for: Targeting UHRF1-SAP30-MXD4 axis for leukemia initiating cell eradication in myeloid leukemia
Source: Cell Res. 2022 Oct 27;32(12):1105–23. doi: 10.1038/s41422-022-00735-6 (PMC9715639; doi:10.1038/s41422-022-00735-6)
Supplement: Supplementary file 3 — Supplementary information Fig 3 [file 41422_2022_735_MOESM3_ESM.pdf]

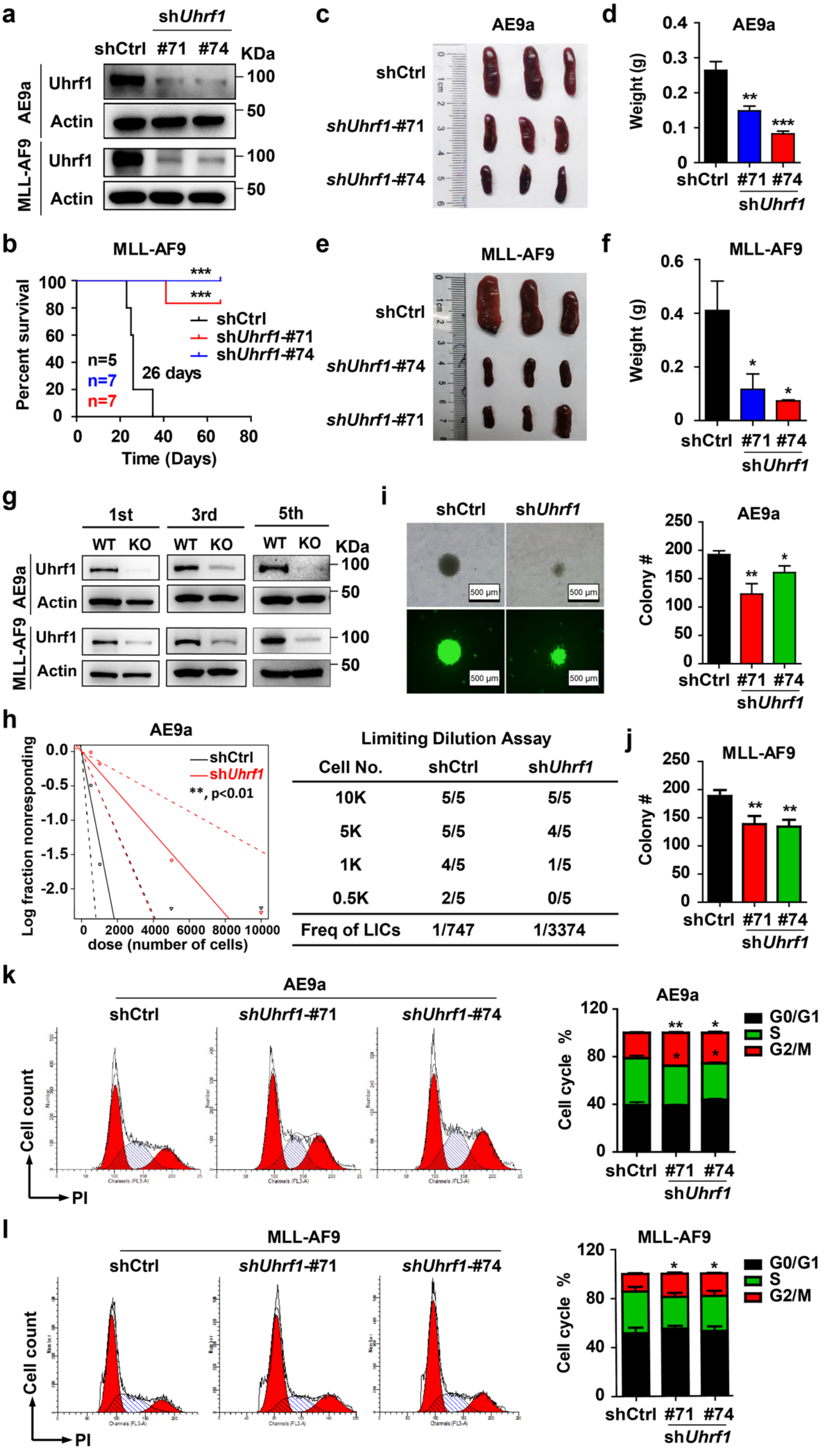
a
shUhrf1
shCtrl #71 #74
KDa
AE9a
Uhrf1
Actin
100
50
MLL-AF9
Uhrf1
Actin
100
50
b
MLL-AF9
Percent survival
100 80 60 40 20 0
***
***
shCtrl
shUhrf1-#71
shUhrf1-#74
n=5
n=7
n=7
26 days
0 20 40 60 80
Time (Days)
c
AE9a
shCtrl
shUhrf1-#71
shUhrf1-#74
d
AE9a
Weight (g)
0.4 0.3 0.2 0.1 0
**
***
shCtrl #71 #74
shUhrf1
e
MLL-AF9
shCtrl
shUhrf1-#74
shUhrf1-#71
f
MLL-AF9
Weight (g)
0.6 0.4 0.2 0
*
*
shCtrl #71 #74
shUhrf1
g
1st 3rd 5th
WT KO WT KO WT KO
KDa
AE9a
Uhrf1
Actin
100
50
MLL-AF9
Uhrf1
Actin
100
50
i
shCtrl shUhrf1
500 µm
AE9a
Colony #
250 200 150 100 50 0
**
*
shCtrl #71 #74
shUhrf1
h
AE9a
Log fraction nonresponding
0.0 -0.5 -1.0 -1.5 -2.0
shCtrl
shUhrf1
**, p<0.01
0 2000 4000 6000 8000 10000
dose (number of cells)
Limiting Dilution Assay
Cell No. | shCtrl | shUhrf1
10K | 5/5 | 5/5
5K | 5/5 | 4/5
1K | 4/5 | 1/5
0.5K | 2/5 | 0/5
Freq of LICs | 1/747 | 1/3374
j
MLL-AF9
Colony #
250 200 150 100 50 0
**
**
shCtrl #71 #74
shUhrf1
k
AE9a
shCtrl shUhrf1-#71 shUhrf1-#74
Cell count
PI
AE9a
Cell cycle %
120 80 40 0
**
*
G0/G1
S
G2/M
shCtrl #71 #74
shUhrf1
l
MLL-AF9
shCtrl shUhrf1-#71 shUhrf1-#74
Cell count
PI
MLL-AF9
Cell cycle %
120 80 40 0
*
*
G0/G1
S
G2/M
shCtrl #71 #74
shUhrf1

Supplementary information Fig. S3 The effects of Uhrf1 inhibition on AML cells and the progression of AML.

a The expression of Uhrf1 was examined by Western blotting analysis in AE9a or MLL-AF9 cells transduced with shRNA against *Uhrf1* or a control shRNA 48 hours after the puromycin selection. **b** The survival of mice that received MLL-AF9 cells expressing shRNA against *Uhrf1* or scrambled shRNA (n≥5). **c-d** The size (**c**) and weight (**d**) of the spleen in mice that received AE9a cells expressing the shRNA against *Uhrf1* or scrambled shRNA 2 weeks after transplantation (n=4). **e-f** The size (**e**) and weight (**f**) of the spleen in the mice that received MLL-AF9 cells expressing shRNA against *Uhrf1* or scrambled shRNA 3 weeks after transplantation (n=4). **g** The expression of Uhrf1 was examined by Western blotting analysis in cells from the colonies generated from AE9a$Uhrf1^{fl/fl}$ CreER or MLL-AF9$Uhrf1^{fl/fl}$ CreER LICs treated with 4-OHT or vehicle. **h** The inhibition of *Uhrf1* decreased the frequency of LICs in the limiting dilution assay. The log-fraction plots showed the result of the limiting dilution assay by using different dilutions of AE9a cells expressing shRNA against *Uhrf1* or scrambled shRNA in vivo. **i-j** The number of colonies generated from AE9a cells (**i**) or MLL-AF9 cells (**j**) transduced with shRNA against *Uhrf1* or scrambled shRNA (n≥3). **k-l** The cell cycle analysis of AE9a cells (**k**) or MLL-AF9 cells (**l**) transduced with shRNA against *Uhrf1* or scrambled shRNA 48 hours after the puromycin selection (n=3). Data are all presented as mean ± SD. Statistical analyses were performed using student's unpaired t-test for **d**, **f**, **i**, **j**, **k** and **l**, and log-rank test for **b**. EDLA software analysis was used for **h**. *p<0.05, **p<0.01, ***p<0.001.
