# Supplementary material for: Targeting UHRF1-SAP30-MXD4 axis for leukemia initiating cell eradication in myeloid leukemia
Source: Cell Res. 2022 Oct 27;32(12):1105–23. doi: 10.1038/s41422-022-00735-6 (PMC9715639; doi:10.1038/s41422-022-00735-6)
Supplement: Supplementary file 4 — Supplementary information Fig 4 [file 41422_2022_735_MOESM4_ESM.pdf]

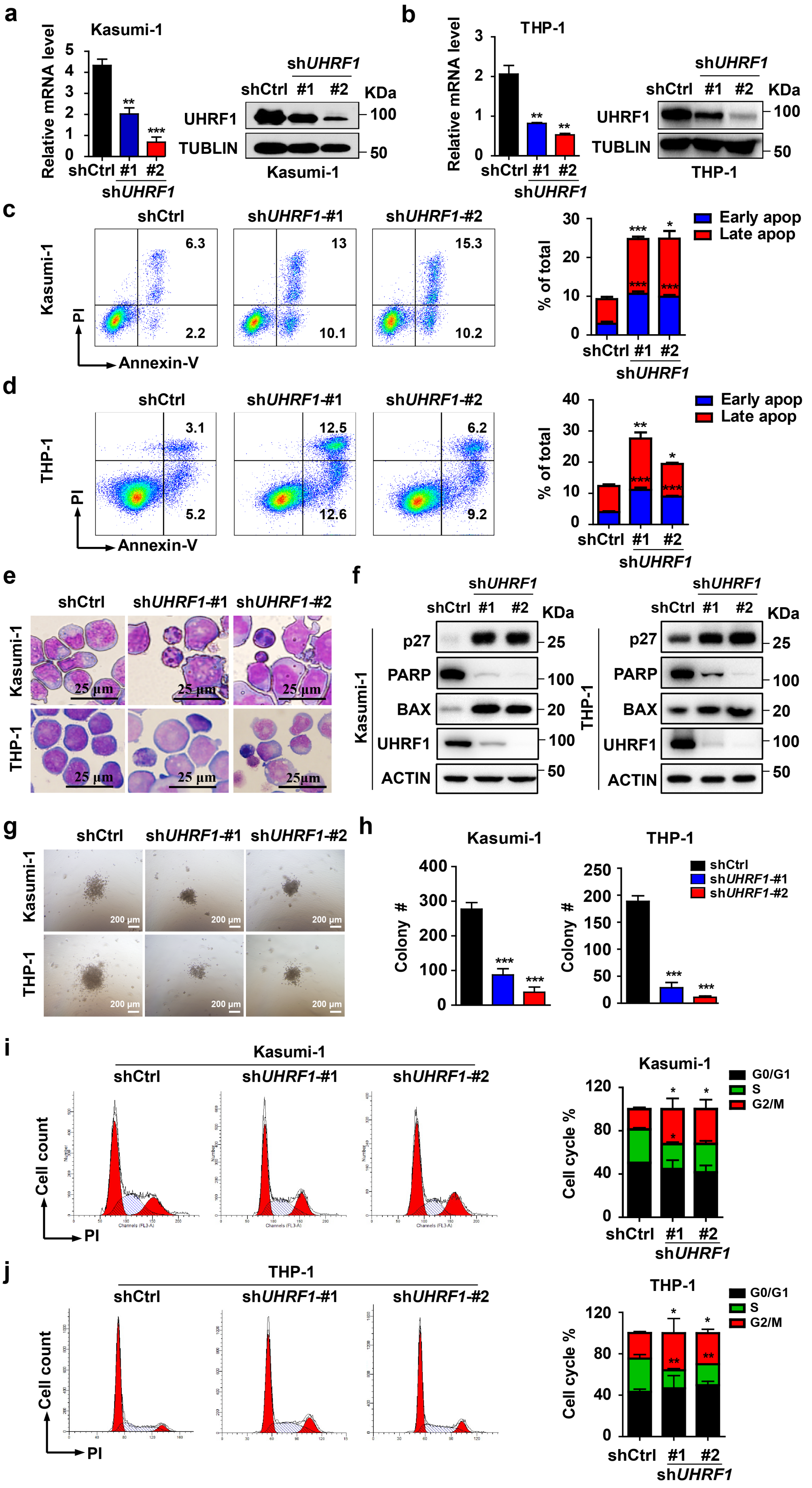

a
Kasumi-1
Relative mRNA level
shCtrl #1 #2
shUHRF1
UHRF1
TUBLIN
KDa
100
50
Kasumi-1
b
THP-1
Relative mRNA level
shCtrl #1 #2
shUHRF1
UHRF1
TUBLIN
KDa
100
50
THP-1
c
shCtrl
shUHRF1-#1
shUHRF1-#2
Kasumi-1
PI
Annexin-V
6.3
2.2
13
10.1
15.3
10.2
% of total
Early apop
Late apop
d
THP-1
3.1
5.2
12.5
12.6
6.2
9.2
e
f
p27
PARP
BAX
UHRF1
ACTIN
g
h
Colony #
shCtrl
shUHRF1-#1
shUHRF1-#2
i
Cell count
Cell cycle %
G0/G1
S
G2/M
j

Supplementary information Fig. S4 The effects of UHRF1 inhibition on the apoptosis, proliferation and cell cycle of human AML cells.

a-b The expression of UHRF1 was examined by q-PCR and Western blotting analysis in Kasumi-1 (**a**) or THP-1 cells (**b**) transduced with the shRNA against *UHRF1* or a control shRNA 48 hours after puromycin selection (n=3). **c-d** The flow cytometry analysis of the apoptosis in Kasumi-1 cells (**c**) or THP-1 cells (**d**) transduced with the shRNA against *UHRF1* or a control shRNA (n≥3). **e** Kasumi-1 and THP-1 cells were transduced with shRNA against *UHRF1* or a control shRNA. The apoptotic cells were identified by the morphological analysis 48 hours after the puromycin selection (scale bar: 25 μM). **f** The Western blotting analysis of p27, PARP, BAX and UHRF1 in Kasumi-1 or THP-1 cells transduced with shRNA against *UHRF1* or a control shRNA. **g-h** The morphology (**g**) and number (**h**) of colonies generated from Kasumi-1 cells or THP-1 cells transduced with shRNA against *UHRF1* or a control shRNA (n=3). **i-j** The cell cycle analysis of Kasumi-1 (**i**) or THP-1 (**j**) cells transduced with shRNA against *UHRF1* or a control shRNA 48 hours after puromycin selection (n=3). Data are all presented as mean ± SD. Statistical analyses were performed using student's unpaired t-test for **a**, **b**, **c**, **d**, **h**, **i** and **j**. *p<0.05, **p<0.01, ***p<0.001.
