# Supplementary material for: Targeting UHRF1-SAP30-MXD4 axis for leukemia initiating cell eradication in myeloid leukemia
Source: Cell Res. 2022 Oct 27;32(12):1105–23. doi: 10.1038/s41422-022-00735-6 (PMC9715639; doi:10.1038/s41422-022-00735-6)
Supplement: Supplementary file 5 — Supplementary information Fig 5 [file 41422_2022_735_MOESM5_ESM.pdf]

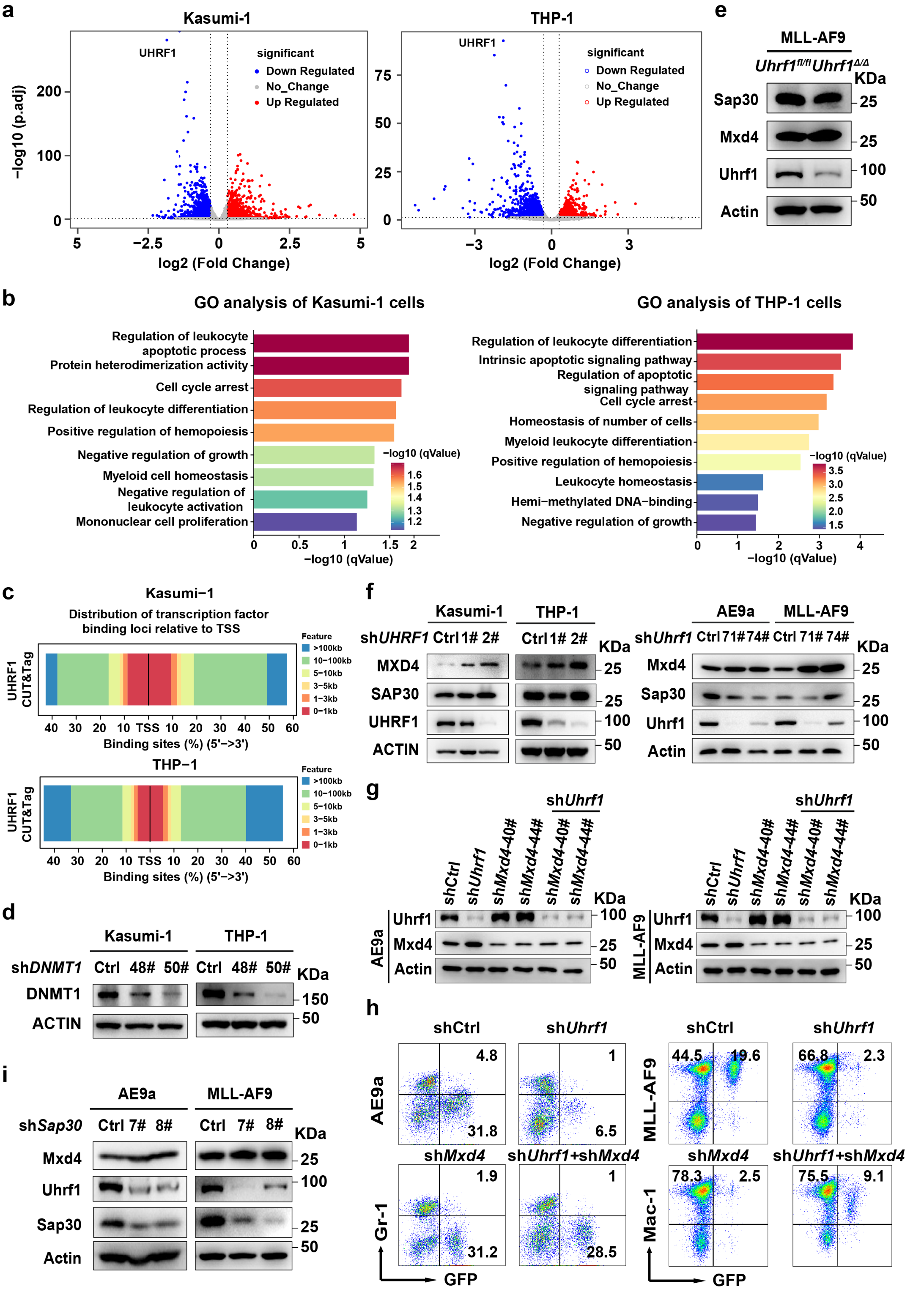

Supplementary information Fig. S5 Transcriptome wide identification of UHRF1 targets in AML Cells.

a The volcano plots of the differential regulated genes in Kasumi-1 or THP-1 cells transduced with the shRNA against *UHRF1* or scrambled shRNA. **b** The gene ontology

analysis of RNA-seq of Kasumi-1 and THP-1 cells transduced with the shRNA against *UHRF1* or scrambled shRNA. **c** The distribution of the transcription factor binding loci relative to the TSS was analyzed by the CUT&Tag of UHRF1 in Kasumi-1 and THP-1 cells. **d** Western blot analysis of DNMT1 expression in AML cells with DNMT1 knockdown. **e** Western blotting analysis of Uhrf1, Sap30 and Mxd4 in murine LICs with Uhrf1 depletion. **f** Western blotting analysis of UHRF1, SAP30 and MXD4 in human and murine AML cells with UHRF1 knockdown. **g** Western blotting analysis of Uhrf1 and Mxd4 expression in AE9a and MLL-AF9 cells with Uhrf1/Mxd4 knockdown. **h** The Gr-1, Mac-1 and GFP in BM cells of the mice that received AE9a or MLL-AF9 cells transduced with the shRNA against *Uhrf1/Mxd4* or scrambled shRNA were examined by the flow cytometry analysis 3 weeks after the transplantation ($n \geq 3$). **i** Western blotting analysis of UHRF1, SAP30 and MXD4 in human and murine AML cells with SAP30 knockdown. Data are all presented as mean ± SD; *$p<0.05$, **$p<0.01$, ***$p<0.001$.
