# Supplementary material for: Targeting UHRF1-SAP30-MXD4 axis for leukemia initiating cell eradication in myeloid leukemia
Source: Cell Res. 2022 Oct 27;32(12):1105–23. doi: 10.1038/s41422-022-00735-6 (PMC9715639; doi:10.1038/s41422-022-00735-6)
Supplement: Supplementary file 6 — Supplementary information Fig 6 [file 41422_2022_735_MOESM6_ESM.pdf]

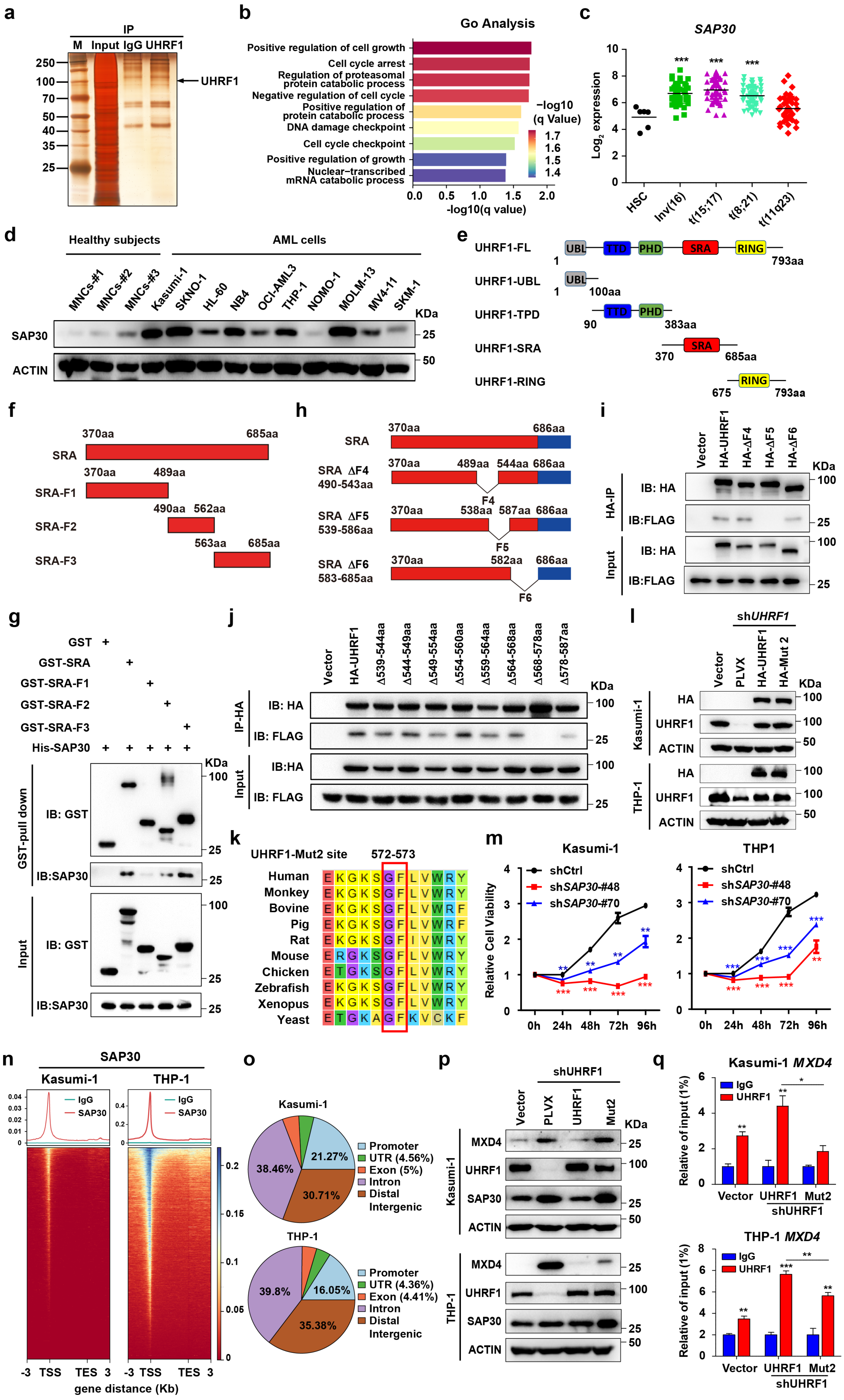
a
IP
M Input IgG UHRF1
250
100
70
50
40
35
25
UHRF1
b
Go Analysis
Positive regulation of cell growth
Cell cycle arrest
Regulation of proteasomal protein catabolic process
Negative regulation of cell cycle
Positive regulation of protein catabolic process
DNA damage checkpoint
Cell cycle checkpoint
Positive regulation of growth
Nuclear-transcribed mRNA catabolic process
−log10 (q Value)
1.7
1.6
1.5
1.4
0.0 0.5 1.0 1.5 2.0
-log10(q value)
c
SAP30
Log2 expression
***
HSC
Inv(16)
t(15;17)
t(8;21)
t(11q23)
d
Healthy subjects
AML cells
MNCs-#1
MNCs-#2
MNCs-#3
Kasumi-1
SKNO-1
HL-60
NB4
OCI-AML3
THP-1
NOMO-1
MOLM-13
MV4-11
SKM-1
KDa
SAP30
ACTIN
e
UHRF1-FL
UBL TTD PHD SRA RING
1 793aa
UHRF1-UBL
1 100aa
UHRF1-TPD
90 383aa
UHRF1-SRA
370 685aa
UHRF1-RING
675 793aa
f
SRA 370aa 685aa
SRA-F1 370aa 489aa
SRA-F2 490aa 562aa
SRA-F3 563aa 685aa
g
GST
GST-SRA
GST-SRA-F1
GST-SRA-F2
GST-SRA-F3
His-SAP30
GST-pull down
IB: GST
IB:SAP30
Input
h
SRA 370aa 686aa
SRA ΔF4 490-543aa
370aa 489aa 544aa 686aa
F4
SRA ΔF5 539-586aa
370aa 538aa 587aa 686aa
F5
SRA ΔF6 583-685aa
370aa 582aa 686aa
F6
i
Vector
HA-UHRF1
HA-ΔF4
HA-ΔF5
HA-ΔF6
HA-IP
IB: HA
IB:FLAG
Input
j
Vector
HA-UHRF1
Δ539-544aa
Δ544-549aa
Δ549-554aa
Δ554-560aa
Δ559-564aa
Δ564-568aa
Δ568-578aa
Δ578-587aa
IP-HA
IB: HA
IB: FLAG
k
UHRF1-Mut2 site 572-573
Human
Monkey
Bovine
Pig
Rat
Mouse
Chicken
Zebrafish
Xenopus
Yeast
l
shUHRF1
Vector
PLVX
HA-UHRF1
HA-Mut 2
Kasumi-1
THP-1
HA
UHRF1
ACTIN
m
Kasumi-1
THP1
shCtrl
shSAP30-#48
shSAP30-#70
Relative Cell Viability
0h 24h 48h 72h 96h
n
SAP30
Kasumi-1
THP-1
IgG
SAP30
-3 TSS TES 3
gene distance (Kb)
o
Kasumi-1
Promoter
UTR (4.56%)
Exon (5%)
Intron
Distal Intergenic
21.27%
38.46%
30.71%
THP-1
UTR (4.36%)
Exon (4.41%)
16.05%
39.8%
35.38%
p
shUHRF1
Vector
PLVX
UHRF1
Mut2
MXD4
UHRF1
SAP30
ACTIN
Kasumi-1
THP-1
q
Kasumi-1 MXD4
THP-1 MXD4
IgG
UHRF1
Relative of input (1%)
Vector UHRF1 Mut2
shUHRF1

Supplementary information Fig. S6 UHRF1 directly interacts with SAP30, which controls *MXD4* transcription.

a The mass spectrometry analysis of UHRF1-interacting proteins in Kasumi-1 cells. Protein gel of immunoprecipitation with anti-UHRF1 antibody is shown. **b** The GO analysis of UHRF1-interacting proteins enriched in the mass spectrometry. **c** The expression of *SAP30* in mononuclear BM or PB cells of AML patients [n-t(15;17)=87, n-Inv(16)=77, n-t(11q23)=88, n-t(8;21)=98)] and the healthy subject controls (n-HSC=6). Data were obtained from the microarray analysis in bloodpool. **d** The expression of SAP30 was examined by Western blotting analysis in various AML cell lines and MNCs from healthy subjects. **e** The schematic representation of UHRF1 and its domains. **f** The schematic representation of the SRA domain and its truncated forms. **g** GST pull-down assay shows that the SRA-F2 and SRA-F3 fragments directly interact with SAP30. **h** The schematic representation of the SRA domain and its truncated forms. **i** The Co-IP assay was performed to examine the interaction of HA-tagged mutant UHRF1 with Flag-SAP30 in 293T cells. **j** The Co-IP assay was performed to examine the interaction of HA-tagged truncated UHRF1 (SRA ΔF5) with Flag-SAP30 in 293T cells (n=3). **k** The alignment of Mut2 (G572, F573) of UHRF1 from human, monkey, bovine, pig, rat, mouse, chicken, zebrafish, xenopus and yeast. **l** The Western blotting analysis of UHRF1 expression in UHRF1 -deficient AML cells after the restoration of HA -tagged UHRF1 and Mut2. **m** The cell viability of Kasumi-1 or THP-1 cells transduced with the shRNA against *SAP30* was examined by MTT assay. **n** The profile and heat-maps of the CUT&Tag peak signals of SAP30 target genes in AML cells. The CUT&Tag analysis was performed by using an antibody against SAP30 and a control IgG in AML cells. **o** The CUT&Tag analysis of the distribution of SAP30 binding sites in AML cells. **P** Western blotting analysis of UHRF1, SAP30 and MXD4 in UHRF1- or Mut2-expressing AML cells with UHRF1 knockdown. **q** ChIP-qPCR analysis of the TSS enrichment of UHRF1 on *MXD4* in UHRF1 or Mut2 (G572R and F573R mutant)-expressing AML cells with UHRF1 knockdown by using the anti-UHRF1 antibody. Data are all presented as mean ± SD; *p<0.05, **p<0.01, ***p<0.001.
