# Supplementary material for: Targeting UHRF1-SAP30-MXD4 axis for leukemia initiating cell eradication in myeloid leukemia
Source: Cell Res. 2022 Oct 27;32(12):1105–23. doi: 10.1038/s41422-022-00735-6 (PMC9715639; doi:10.1038/s41422-022-00735-6)
Supplement: Supplementary file 7 — Supplementary information Fig 7 [file 41422_2022_735_MOESM7_ESM.pdf]

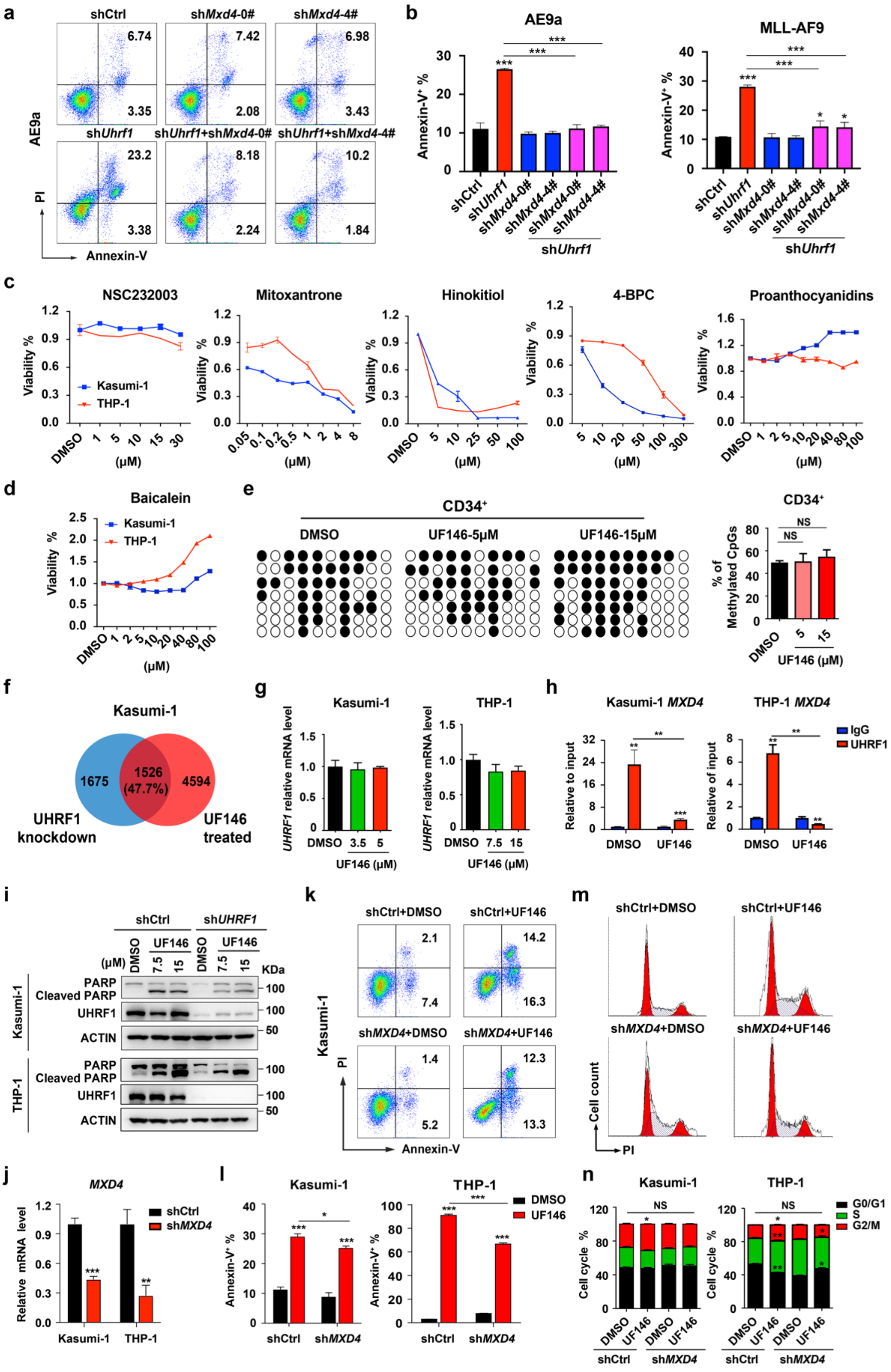

o

GSEA analysis of Kasumi-1 with UHRF1 Knockdown

	GS follow link to MsigDB	NES	FDR q-val
1	HALLMARK_MYC_TARGETS_V2	2.12	0
2	HALLMARK_E2F TARGETS	2.06	0
3	HALLMARK_MYC_TARGETS_V1	2.04	0
4	HALLMARK_G2M_CHECKPOINT	1.92	0.001
5	HALLMARK_UNFOLDED_PROTEIN_RESPONSE	1.72	0.004
6	HALLMARK_MTORC1_SIGNALING	1.68	0.005

GSEA analysis of THP-1 with UHRF1 Knockdown

	GS follow link to MsigDB	NES	FDR q-val
1	HALLMARK_MYC_TARGETS_V1	1.97	0.002
2	HALLMARK_E2F TARGETS	1.93	0.001
3	HALLMARK_MYC_TARGETS_V2	1.88	0.001
4	HALLMARK_MTORC1_SIGNALING	1.85	0
5	HALLMARK_UNFOLDED_PROTEIN_RESPONSE	1.75	0.002
6	HALLMARK_G2M_CHECKPOINT	1.67	0.003

Supplementary information Fig. S7 The targets of UF146 on human AML cells.

a-b The representative flow cytometry profiles (**a**) and quantification (b) of apoptosis in AE9a and MLL-AF9 cells with Uhrf1/Mxd4 knockdown. **c-d** MTT analysis in Kasumi-1 and THP-1 cells with the treatment of other UHRF1 inhibitors. Some inhibitors didn't affect the proliferation of AML cells (NSC232003, Proanthocyanidins and Baicalein) (**c**, **d**), and others inhibited the proliferation of AML cells (Mitoxantrone, Hinokitiol and 4-BPC) (**c**). **e** The DNA methylation analysis of *MXD4* by the bisulfite sequencing in UF146 or vehicle-treated human $CD34^+$ cells isolated from cord blood. **f** Venn diagram analysis using differential expressed genes upregulated in Kasumi-1 cells after treatment of UF146 and those after UHRF1 knockdown relative to mock. **g** qPCR analysis of UHRF1 in Kasumi-1 and THP-1 cells 24h after UF146 treatment. **h** ChIP-qPCR analysis of UHRF1 enrichment on *MXD4* promoter in AML cells 24 hours after UF146 treatment by using the anti-UHRF1 antibody. **i** Western blotting analysis of the apoptosis-related protein in UF146-treated Kasumi-1 and THP-1 cells with UHRF1 knockdown. **j** qPCR analysis of MXD4 in AML cells with MXD4 knockdown. **k-n** The apoptosis **(k-l)** and cell cycle **(m-n)** analysis of UF146 (3.5 μM)-treated Kasumi-1 cells and UF146 (7.5 μM)-treated THP-1 cells transduced with the shRNA against MXD4 or scrambled shRNA 48 hours after puromycin selection. **o** List of GSEA analysis of RNA-seq data in Kasumi-1 and THP-1 cells with UHRF1 knockdown. Statistical analyses were performed using student's unpaired t-test for **b**, **e**, **h**, **j**, **l** and **n**. NS meas no significant differences. *p<0.05, **p<0.01, ***p<0.001.
