# Supplementary material for: Targeting UHRF1-SAP30-MXD4 axis for leukemia initiating cell eradication in myeloid leukemia
Source: Cell Res. 2022 Oct 27;32(12):1105–23. doi: 10.1038/s41422-022-00735-6 (PMC9715639; doi:10.1038/s41422-022-00735-6)
Supplement: Supplementary file 8 — Supplementary information Fig 8 [file 41422_2022_735_MOESM8_ESM.pdf]

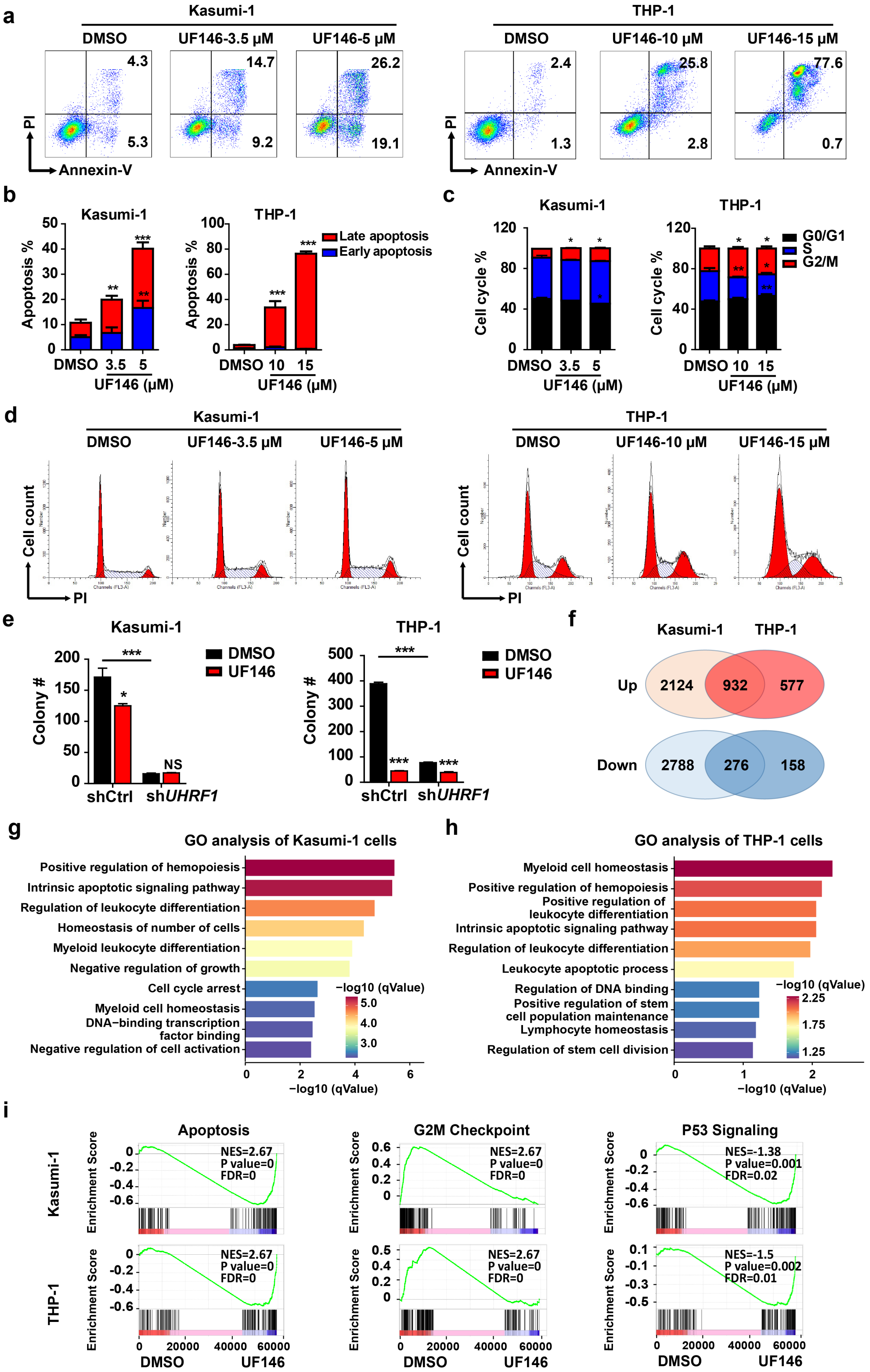

Supplementary information Fig. S8 The effects of UF146 on human AML cells.

a-b The representative flow cytometry profiles (**a**) and quantification (**b**) of apoptosis

was examined by flow cytometry analysis in Kasumi-1 and THP-1 cells 24 hours after the treatment of UF146 or the vehicle control (n=3). **c-d** The representative flow cytometry profiles (**d**) and quantification (**c**) of cell cycle analysis of Kasumi-1 and THP-1 cells 24 hours after the treatment of UF146 or the vehicle control (n=3). **e** The number of colonies generated by Kasumi-1 or THP-1 cells treated with UF146 or the vehicle control after knockdown of *UHRF1* (n=3). **f** The overlap of the differential expressed genes in RNA-seq of Kasumi-1 and THP-1 cells treated with UF146 or the vehicle control. **g-h** The gene ontology analysis of overlapped genes in RNA-seq of Kasumi-1(**g**) and THP-1(**h**) cells treated with UF146 or the vehicle control. **i** The GSEA analysis of the apoptosis, G2M checkpoint and p53 pathways in Kasumi-1 and THP-1 cells treated with UF146. Data are all presented as mean ± SD. Statistical analyses were performed using student's unpaired t-test for **b**, **c** and **e**. *p<0.05, **p<0.01, ***p<0.001.
