# Supplementary material for: Targeting UHRF1-SAP30-MXD4 axis for leukemia initiating cell eradication in myeloid leukemia
Source: Cell Res. 2022 Oct 27;32(12):1105–23. doi: 10.1038/s41422-022-00735-6 (PMC9715639; doi:10.1038/s41422-022-00735-6)
Supplement: Supplementary file 9 — Supplementary information Fig 9 [file 41422_2022_735_MOESM9_ESM.pdf]

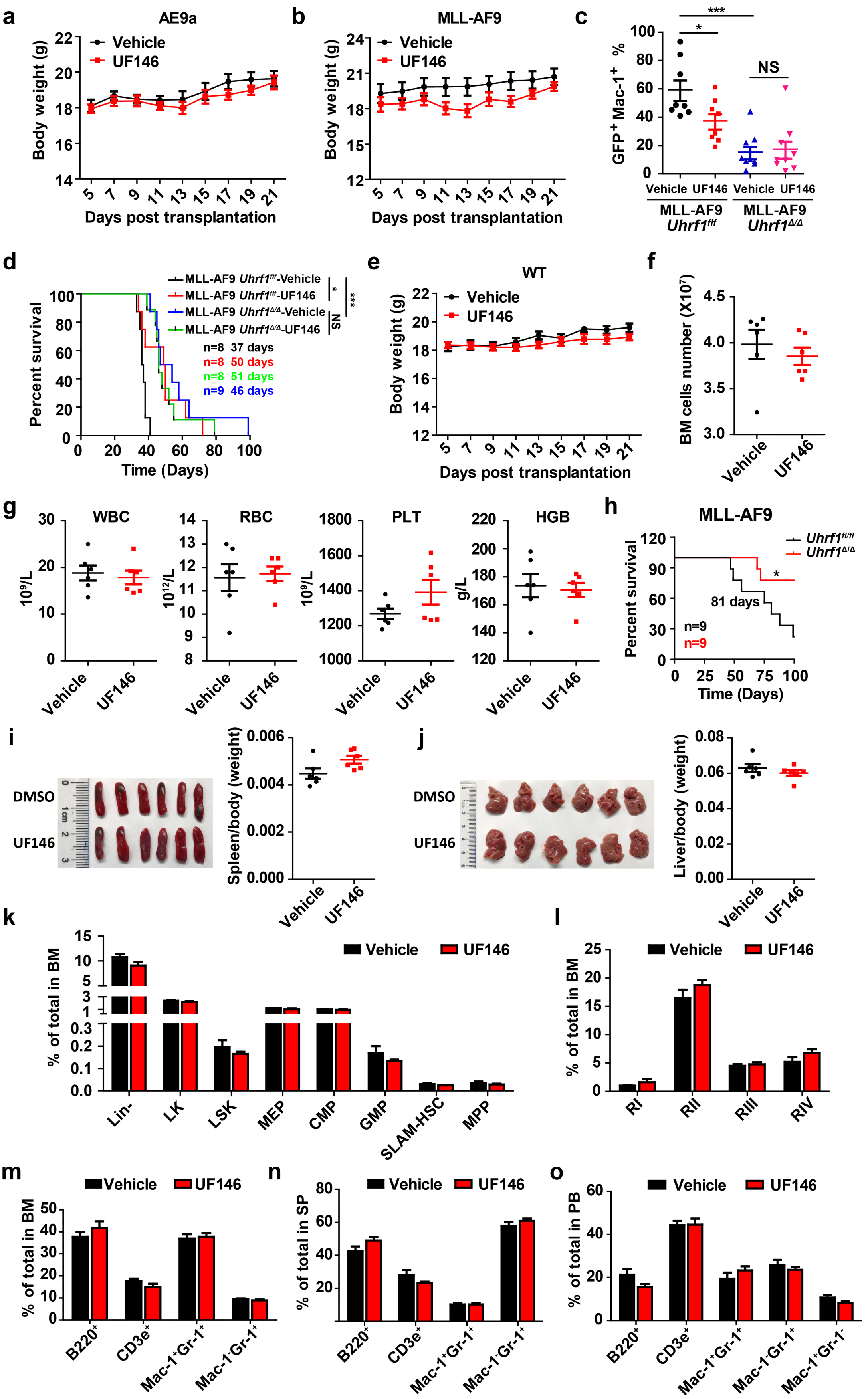

a
AE9a
Vehicle
UF146
Body weight (g)
Days post transplantation
b
MLL-AF9
Vehicle
UF146
Body weight (g)
Days post transplantation
c
GFP⁺Mac-1⁺ %
***
*
NS
Vehicle UF146 Vehicle UF146
MLL-AF9 Uhrf1f/f
MLL-AF9 Uhrf1Δ/Δ
d
MLL-AF9 Uhrf1f/f-Vehicle
MLL-AF9 Uhrf1f/f-UF146
MLL-AF9 Uhrf1Δ/Δ-Vehicle
MLL-AF9 Uhrf1Δ/Δ-UF146
n=8 37 days
n=8 50 days
n=8 51 days
n=9 46 days
Percent survival
Time (Days)
e
WT
Vehicle
UF146
Body weight (g)
Days post transplantation
f
BM cells number (X10⁷)
Vehicle
UF146
g
WBC
RBC
PLT
HGB
h
MLL-AF9
Uhrf1fl/fl
Uhrf1Δ/Δ
81 days
n=9
n=9
Percent survival
Time (Days)
i
DMSO
UF146
Spleen/body (weight)
j
DMSO
UF146
Liver/body (weight)
k
Vehicle
UF146
% of total in BM
Lin- LK LSK MEP CMP GMP SLAM-HSC MPP
l
Vehicle
UF146
% of total in BM
RI RII RIII RIV
m
Vehicle
UF146
% of total in BM
B220⁺ CD3e⁺ Mac-1⁺Gr-1⁺ Mac-1⁻Gr-1⁺
n
Vehicle
UF146
% of total in SP
B220⁺ CD3e⁺ Mac-1⁺Gr-1⁺ Mac-1⁻Gr-1⁺
o
Vehicle
UF146
% of total in PB
B220⁺ CD3e⁺ Mac-1⁺Gr-1⁺ Mac-1⁻Gr-1⁺ Mac-1⁺Gr-1⁻

Supplementary information Fig. S9 UF146 has minimal effects on normal hematopoiesis.

a-b The body weight of AE9a (**a**) and MLL-AF9 (**b**) mice treated with UF146 (2.5 mg/kg) or the vehicle control (n≥6). **c** The flow cytometry analysis of $GFP^{+}Mac\text{-}1^{+}$ cells in PB of MLL-AF9*Uhrf1*$^{fl/fl}$ mice or MLL-AF9*Uhrf1*$^{\Delta/\Delta}$ mice treated with UF146 or the vehicle control 3 weeks after the transplantation (n≥8). **d** The survival of MLL-AF9*Uhrf1*$^{fl/fl}$ mice or MLL-AF9*Uhrf1*$^{\Delta/\Delta}$ mice treated with UF146 or the vehicle control (n≥8). **e** The body weight of WT mice treated with UF146 (2.5 mg/kg) or the vehicle control (n≥6). **f-g** The C57 mice were treated with UF146 (2.5 mg/kg) or the vehicle control for three weeks (n=6). The numbers of total BM cells were counted (f), and the CBC analysis was performed (g) (n=6). **h** The survival curve of MLL-AF9Uhrf1 $^{fl/fl}$ mice or MLL-AF9Uhrf1$^{\Delta/\Delta}$ mice in AML initiation experiment. **i-j** The C57 mice were treated with UF146 (2.5 mg/kg) or the vehicle control for three weeks (n=6). The size and weight of the spleen (**i**) and liver (**j**) were shown. **k-l** C57 mice were treated with UF146 (2.5 mg/kg) or the vehicle control for three weeks. The flow cytometry analysis of hematopoietic stem and progenitor populations (**k**) and erythroid differentiation populations (**l**) in BM cells was performed (n=6). **m-o** The C57 mice were treated with UF146 (2.5 mg/kg) or the vehicle control for three weeks. The flow analysis of mature hematopoietic populations in the BM (**m**), spleen (**n**), and PB (**o**) from these mice (n=6) was performed (n=6). Data are all presented as mean ± SD. Statistical analyses were performed using student's unpaired t-test for **a**, **b**, **c**, **e**, **f**, **g** and **i-o**, and log-rank test for **d**, **h**.*$p<0.05$, **$p<0.01$, ***$p<0.001$.
