# Supplementary material for: Targeting UHRF1-SAP30-MXD4 axis for leukemia initiating cell eradication in myeloid leukemia
Source: Cell Res. 2022 Oct 27;32(12):1105–23. doi: 10.1038/s41422-022-00735-6 (PMC9715639; doi:10.1038/s41422-022-00735-6)
Supplement: Supplementary file 10 — Supplementary information Fig 10 [file 41422_2022_735_MOESM10_ESM.pdf]

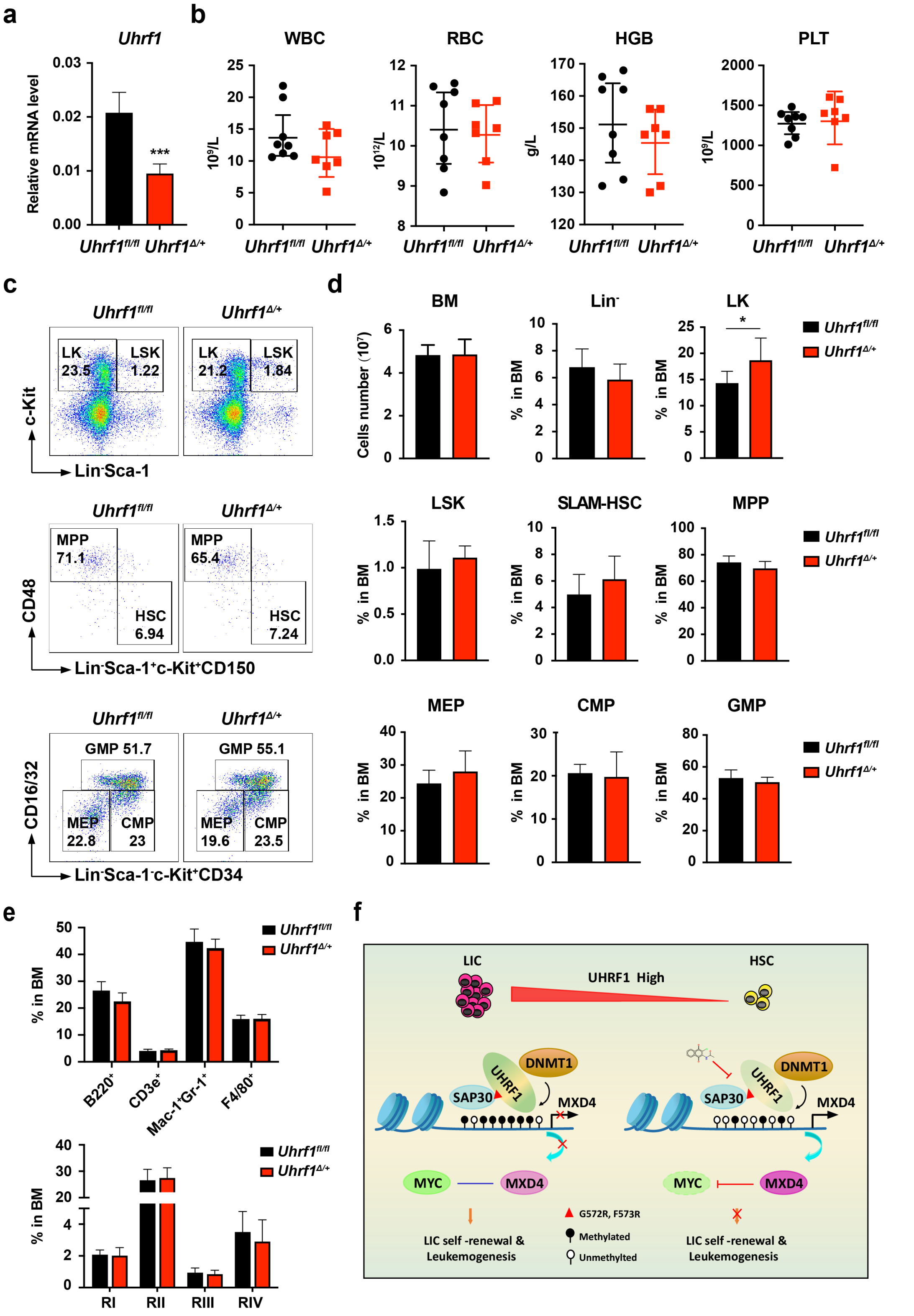

Supplementary information Fig. S10. The effect of Uhrf1 heterozygous knockout on hematopoiesis in mice. (a) The mRNA level of Uhrf1 in BM cells isolated from *Uhrf1*$^{fl/fl}$ and *Uhrf1*$^{\Delta/+}$ mice with poly(I:C) treatment. **(b)** The WBC count analysis of *Uhrf1*$^{fl/fl}$ and *Uhrf1*$^{\Delta/+}$ mice. **(c-d)** The number of total BM cells was counted **(d)** and

flow cytometry analysis of hematopoietic stem and progenitor populations **(c-d)** in BM cells was performed. **(e)** The flow cytometry analysis of multilineage differentiated blood cells in BM was performed. **(f)** Schematic overview of AML gene regulation by Uhrf1. Statistical analyses were performed using student's unpaired t-test for **a**, **b**, **d** and **e**. *p<0.05, **p<0.01, ***p<0.001.
